# Enhancing interpretability of AI with radiomics-based deep neural network: proof of concept in the classification of Parkinsonian syndromes with ^18^F-FDG PET imaging

**DOI:** 10.1007/s00259-025-07478-7

**Published:** 2025-09-11

**Authors:** Chenyang Li, Fangyang Jiao, Shaoyou Wu, Chenhan Wang, Min Wei, Shuoyan Zhang, Luyao Wang, Yu Huang, Yafu Yin, Rong Tian, Alexander Bernhardt, Sabrina Katzdobler, Johannes Levin, Günter U. Höglinger, Matthias Brendel, Axel Rominger, Kuangyu Shi, Chuantao Zuo, Jiehui Jiang

**Affiliations:** 1 Institute of Biomedical Engineering, School of Life Sciences, Shanghai University, Shanghai, 200444 China; 2 Department of Nuclear Medicine, The First Affiliated Hospital of Chongqing Medical University, Chongqing, China; 3 Department of Neurology, Beijing Friendship Hospital, Capital Medical University, Beijing, China; 4 Department of Neurology, Xuanwu Hospital of Capital Medical University, Beijing, China; 5 School of Communication and Information Engineering, Shanghai University, Shanghai, China; 6 Department of Nuclear Medicine, Xinhua Hospital, Shanghai Jiao Tong University School of Medicine, Shanghai, China; 7 Department of Nuclear Medicine, West China Hospital, Sichuan University, Sichuan, China; 8 Department of Neurology, University Hospital, LMU Munich, Munich, Germany; 9 German Center for Neurodegenerative Diseases (DZNE), Munich, Germany; 10 Munich Cluster for Systems Neurology (SyNergy), Munich, Germany; 11 Department of Nuclear Medicine, University Hospital, LMU Munich, Munich, Germany; 12 Department of Nuclear Medicine, University Hospital Bern, Bern, Switzerland; 13 Computer Aided Medical Procedures, School of Computation, Information and Technology, Technical University of Munich, Munich, Germany; 14 Human Phenome Institute, Fudan University, Shanghai, China; 15 Department of Nuclear Medicine & PET Center, Huashan Hospital, Fudan University, Shanghai, 200235 China; 16 Department of Nuclear Medicine, The First Affiliated Hospital of Chongqing Medical Univers, Chongqing, 400010 China

**Keywords:** Model interpretability, Radiomics, Dual-channel neural network, Parkinsonian syndromes, ^18^F-fluorodeoxyglucosePET imaging, SHAP

## Abstract

**Objective:**

Interpretability and reproducibility remain major challenges in applying deep neural network (DNN) to neuroimaging-based diagnosis. This study proposes a radiomics-guided dual-channel deep neural network (RDDNN) to improve feature transparency and enhance clinical understanding in the classification of Parkinsonian syndromes.

**Methods:**

In this bi-centric study, we analysed two independent cohorts comprising 1,275 patients with idiopathic Parkinson’s disease (IPD), multiple system atrophy (MSA), and progressive supranuclear palsy (PSP), alongside 223 healthy controls from Huashan Hospital and 90 patients with IPD, MSA, and PSP (34IPD, 17MSA, 39PSP) from the University Hospital Munich. It is a re-analysis of well-studied Chinese and German cohorts of ^18^F-fluorodeoxyglucose Positron emission tomography (FDG-PET) imaging of parkinsonian patients and the FDG scans were of 10-min static acquisition at 60 min post FDG injection and normalized against whole brain activity. The RDDNN model combines local features extracted via dilated convolutional networks and global features derived from Transformer-based self-attention networks. Model performance was evaluated using classification metrics and compared to radiomics and DNN approaches. The model’s outputs were also compared with nuclear medicine specialists’ visual assessments to assess interpretability and time efficiency. Furthermore, SHapley Additive Explanations (SHAP), Layer-wise Class Activation Mapping (Layer-CAM), and Rollout Attention Map (RAM) were employed to evaluate which features played the most critical roles in the model’s final classification decisions after supervised training, and to examine how both networks spatially corresponded to known brain connectivity regions.

**Results:**

In the internal blind-test cohort, the RDDNN achieved high accuracy (AUC = 0.99, accuracy = 0.98). SHAP and correlation analyses jointly indicated complementary information across channels, some of which were clinically interpretable. In the external cohort, the model maintained robust performance (AUC = 0.94, accuracy = 0.81), with consistent feature patterns across populations. The model significantly reduced evaluation time compared to nuclear medicine specialists’ readings (*p* < 0.001), and the heatmaps showed disease-specific activation in anatomically relevant regions for IPD, MSA, and PSP.

**Conclusion:**

The RDDNN framework provides a clinically interpretable and reproducible DNN solution for classifying Parkinsonian disorders. By integrating radiomics and attention-based modeling, it enhances lesion localization, supports clinical decision-making, and offers performance comparable to human specialists—while substantially improving diagnostic efficiency.

**Supplementary Information:**

The online version contains supplementary material available at 10.1007/s00259-025-07478-7.

## Introduction

Atypical parkinsonian syndromes (APS), including multiple system atrophy (MSA) and progressive supranuclear palsy (PSP), are a group of neurodegenerative disorders that clinically resemble idiopathic Parkinson’s disease (IPD) in their early stages. However, they differ significantly with IPD in etiology, progression, and treatment response [1]. Due to overlapping motor symptoms in the prodromal phase—such as bradykinesia, rigidity, and postural instability—early differentiation between IPD and APS remains challenging and is a common cause of misdiagnosis. For example, MSA often presents with parkinsonism accompanied by autonomic dysfunction and cerebellar signs [2], while PSP typically features gait freezing, vertical gaze palsy, and early-onset dysphagia and dysarthria, which help differentiate it from other forms of Parkinsonism [3]. Given these complexities, the development of accurate and reliable biomarkers is critical for distinguishing between these conditions and enabling more individualized therapeutic decision-making.

Positron emission tomography (PET) has been widely utilized in neuroimaging to aid the diagnosis and differentiation of parkinsonian syndromes, particularly distinguishing IPD from atypical variants such as PSP and MSA [4]. Among available radiotracers, ^18^F-fluorodeoxyglucose PET (FDG-PET) is particularly effective for capturing disease-related metabolic changes [5]. Several studies have sought to improve diagnostic accuracy by analyzing metabolic patterns and developing visual assessment tools [6–9]. In parallel, artificial intelligence (AI) techniques—particularly radiomics and deep learning (DL)—have gained traction in FDG-PET image analysis [10]. For instance, Sun et al. [11]. developed a multimodal PET/MRI radiomics model to distinguish IPD from MSA, achieving an AUC of 0.919 in the validation cohort; while another study by Sun et al. [12] applied a deep learning radiomics framework on FDG-PET to differentiate IPD patients from healthy controls(HCs), reporting an accuracy of 89.3% and AUC of 0.947 in the independent test set. Similarly, Wu et al. [13]. exhibited outstanding performance(sensitivities of 88.5% for MSA, and 84.5% for PSP) in distinguishing the metabolic markers of APS through their DL model.

Despite these advances, interpretability and reproducibility remain major limitations. Radiomics models often rely on manual region-of-interest segmentation, introducing human bias and limiting scalability [14, 15]. Traditional DL models excel at local feature extraction but often lack spatial context, leading to poor lesion-level explainability across the brain [16, 17]. Furthermore, existing AI frameworks rarely provide anatomically plausible decision pathways—that is, consistent attribution of model decisions to clinically recognized, disease-relevant brain regions [18]—nor do they ensure feature stability across cohorts.

To address these diagnostic and interpretability challenges, we propose a radiomics-guided dual deep neural network (RDDNN) to differentiate IPD, MSA and PSP using brain FDG-PET imaging. The model is built with two complementary processing pathways: one focuses on local metabolic changes using dilated deep neural network (DNN) networks, while the other captures global patterns through a Transformer network. To enhance clinical trust, we introduce three levels of interpretability: (1) Feature integration across radiomics and AI-extracted patterns; (2) Quantification of key feature importance using an explainable SHapley Additive Explanations (SHAP) value; (3) Visualization of disease-specific brain regions using attention-based mapping techniques that align well with known metabolic abnormalities in MSA and PSP.

## Materials and methods

### Subjects

In this study, we recruited a total of 1,498 subjects from Huashan Hospital, Fudan University, based on FDG-PET imaging scans, including 223 HCs and 1,275 patients with Parkinsonism (751 with IPD, 290 with MSA, and 234 with PSP). The diagnoses of patients were made based on the corresponding criteria for IPD, MSA, and PSP [19–21]. Variables collected were sex, age (years), disease duration and fellow-up (months), and MDS Unified Parkinson’s Disease Rating Scale III(UPDRSIII) (Med-off).

**Fig. 1:**
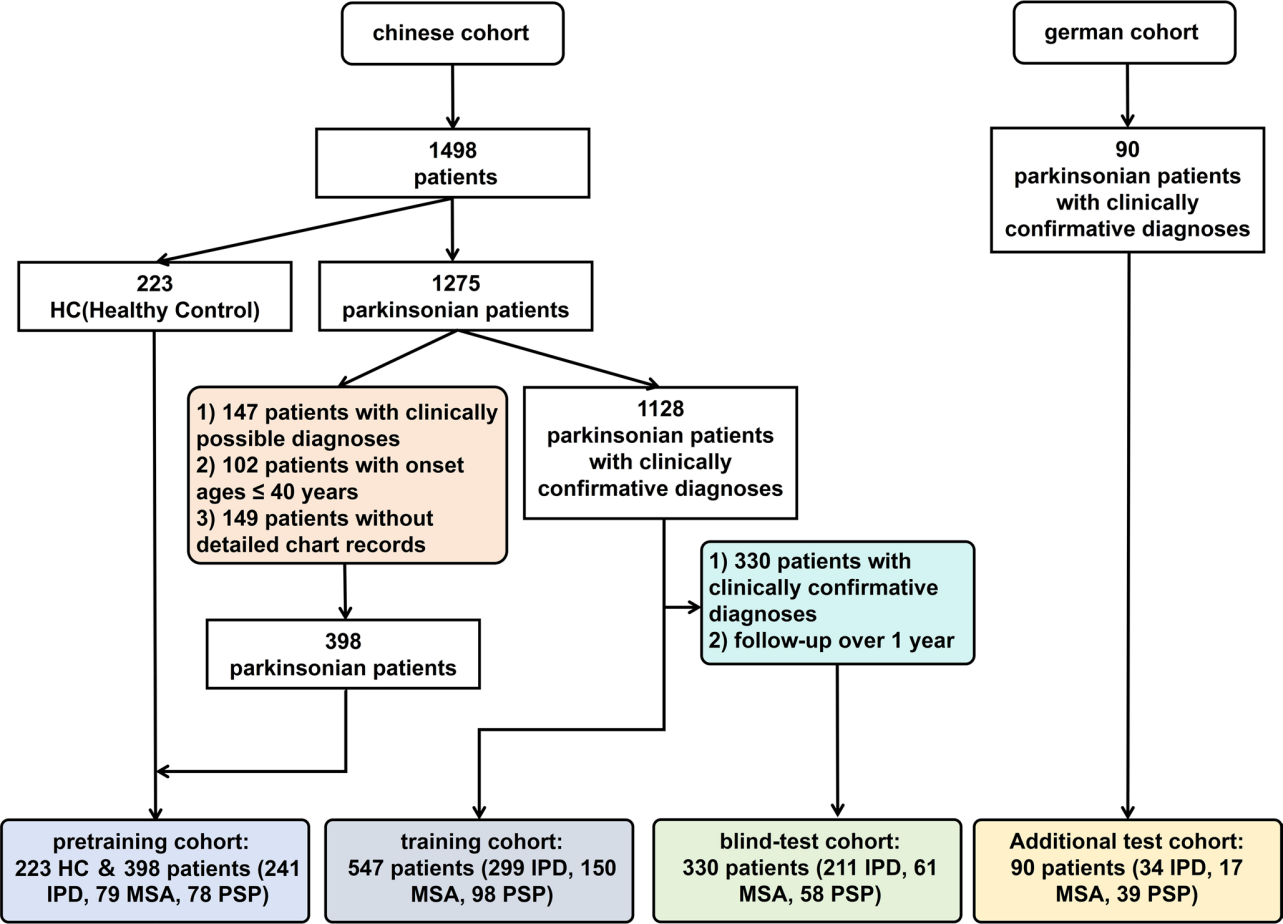
A Chinese cohort, utilizing the Huashan Parkinsonian PET imaging dataset, with a German cohort

The cohort was divided into three parts for analysis: the pretraining cohort, which included 220 HCs and 398 patients (comprising 241 with IPD, 79 with MSA, and 78 with PSP); the training cohort, consisting of 547 clearly diagnosed patients (299 with IPD, 150 with MSA, and 98 with PSP); and the blind-test cohort, which included 330 confirmed and followed-up patients (211 with IPD, 61 with MSA, and 58 with PSP). The division criteria were based on diagnosis classifications: ‘clinically probable,’ ‘clinically definite,’ and ‘clinically confirmed.’ ‘clinically probable,’ and ‘clinically definite,’ were made according to corresponding criteria by clinical experts, whereas a clinically confirmed diagnosis relied on at least one formal clinical follow-up over one year after PET imaging. Low-dose CT was employed for attenuation correction, and emission scans were obtained 60 min after the injection of approximately 185 MBq ± 18.5 of ^18^F-FDG, lasting 10 min (Siemens Biograph 64 HD PET/CT, Siemens Healthcare, Erlangen, Germany). After corrections for attenuation, scatter, dead time, and random coincidences, PET images were reconstructed using the ordered subset expectation maximization (OSEM) method. Routine MRI examinations were performed before PET scans and those patients with structural brain abnormalities were excluded. After PET examination, patients had at least one return visit and the movement disorders specialists made a clinical diagnosis according to the latest clinical criteria [19–21]. The division of the different cohorts is illustrated in Fig. 1. For detailed information of the Chinese Cohort, please refer to Supplementary Material 1.

Additionally, for further independent testing and external validation, we recruited 90 Parkinsonian patients from Germany at the University Hospital of Munich, consisting of 34 with IPD, 17 with MSA, and 39 with PSP, all clinically diagnosed. The imaging scans were conducted using various PET/CT systems (ECAT Exact HR+ [Siemens], Discovery 690 [GE Healthcare], and Biograph 64) according to the European Association of Nuclear Medicine protoco l [22] using a slow bolus injection of approximately 150 MBq of ^18^F-FDG for slow intravenous injection. For data difference between Chinese and German Cohort, please refer to Supplementary Material 2 and Table S2.

The Institutional Review Board granted and approved the data analysis and ethical approval for this study (from Huashan Hospital [identifier: KY2011-174, date: August 24, 2011] [13] and the University of Munich [23, 24]), with informed consent obtained from all subjects.

### PET image acquisition and reconstruction

FDG-PET images were normalized to a PET brain template in Montreal Neurological Institute (MNI) space using SPM12 software implemented in MATLAB 9.11.0 (Mathworks Inc., Sherborn, MA, USA) [25]. Subsequently, the normalized PET images were smoothed with a three-dimensional Gaussian filter with a full width at half maximum (FWHM) of 10 mm. The standardized uptake value ratio (SUVR) was used to measure local glucose metabolic activity normalized to overall activity [26]. PET images were standardized using z-score normalization before being input into the DNN to improve training stability.

### RDDNN model workflow

**Fig. 2:**
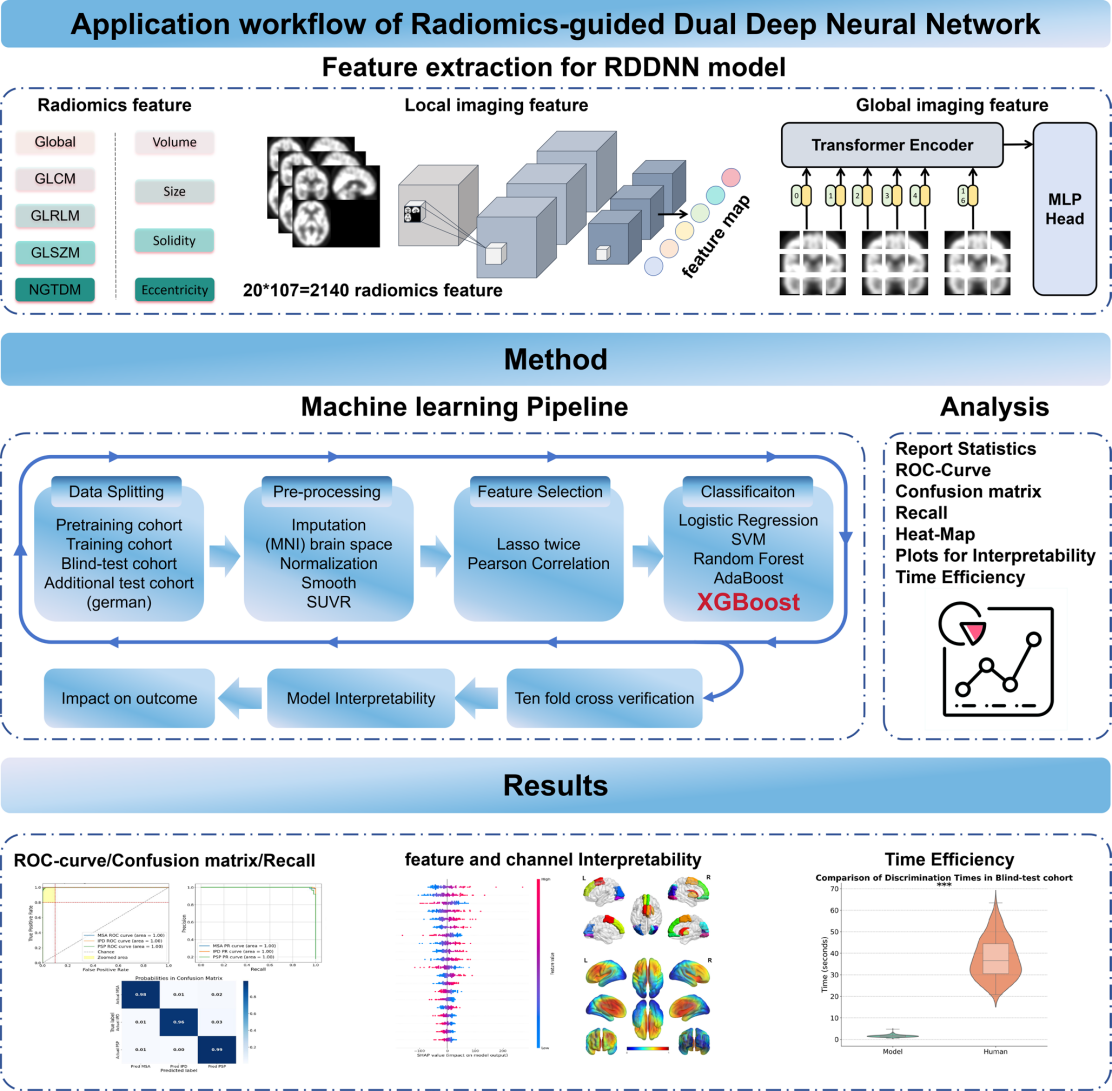
Workflow of the RDDNN model. This diagram illustrates the process of feature extraction and classification within the RDDNN framework, highlighting the integration of dual-channel input from FDG-PET images. The workflow includes stages such as radiomics feature extraction, processing through the local and global subnetworks, feature fusion, and final classification outcomes

The dual-channel design is grounded in the inherent structural priors and feature extraction strengths of the underlying architectures. CNNs are well-suited for extracting spatially localized, low-level features, such as texture patterns and metabolic gradients, due to their limited receptive fields and strong local connectivity assumptions [27, 28]. In contrast, Transformer-based models excel at modeling global contextual dependencies and capturing long-range inter-regional relationships across the input space through their self-attention mechanism [29, 30]. By integrating these two complementary streams [31], the RDDNN framework aims to comprehensively characterize both focal and distributed metabolic abnormalities, which is critical in diseases like Parkinsonism that exhibit substantial spatial heterogeneity in pathological manifestation. Fig. 2 shows the workflow of the RDDNN Model. The model utilizes preprocessed PET images as input and incorporates various networks within an ensemble framework that combines radiomics features. This design enables the construction of a dual-channel feature fusion network that integrates both local and global feature channel networks. The workflow can be summarized into four key components: (1) data feature preparation for the RDDNN model; (2) methodological workflow; (3) data analysis; (4) model classification, interpretability, reproducibility and efficiency.

#### Radiomics feature extraction

Using the open-source Pyradiomics version 3.1.0 software [32], radiomics features were extracted from the FDG PET images in the pretraining and training groups. To delineate the regions of interest (ROIs) [33], we utilized both the Automated Anatomical Labeling (AAL3) atlas and the PD25 atlas [34]. The analysis focused on 20 specific areas: frontal cortex, parietal cortex, occipital cortex, temporal cortex, subthalamic nucleus, nucleus accumbens, ventral tegmental area, locus coeruleus, raphe nucleus, dentate nucleus, substantia nigra, red nucleus, thalamic basal nucleus, caudate nucleus, putamen, globus pallidus, thalamus, medulla oblongata, midbrain, and pons. From each ROI, a total of 107 radiomics features were extracted, resulting in an overall sum of 2,140 features (The descriptions about all 107 radiomics features were in Table S3 of the Supplementary Materials 3). These features encompassed various dimensions, including shape, glucose uptake distribution, and texture metrics derived from different matrices: the gray-level co-occurrence matrix (GLCM), gray-level run-length matrix (GLRLM), gray-level size zone matrix (GLSZM), and edge features from the neighborhood gray tone difference matrix (NGTDM). Each radiomics feature was normalized using Z-scores calculated based on the mean and standard deviation of feature values in the training cohort.

#### Dual deep neural network feature extraction

**Fig. 3:**
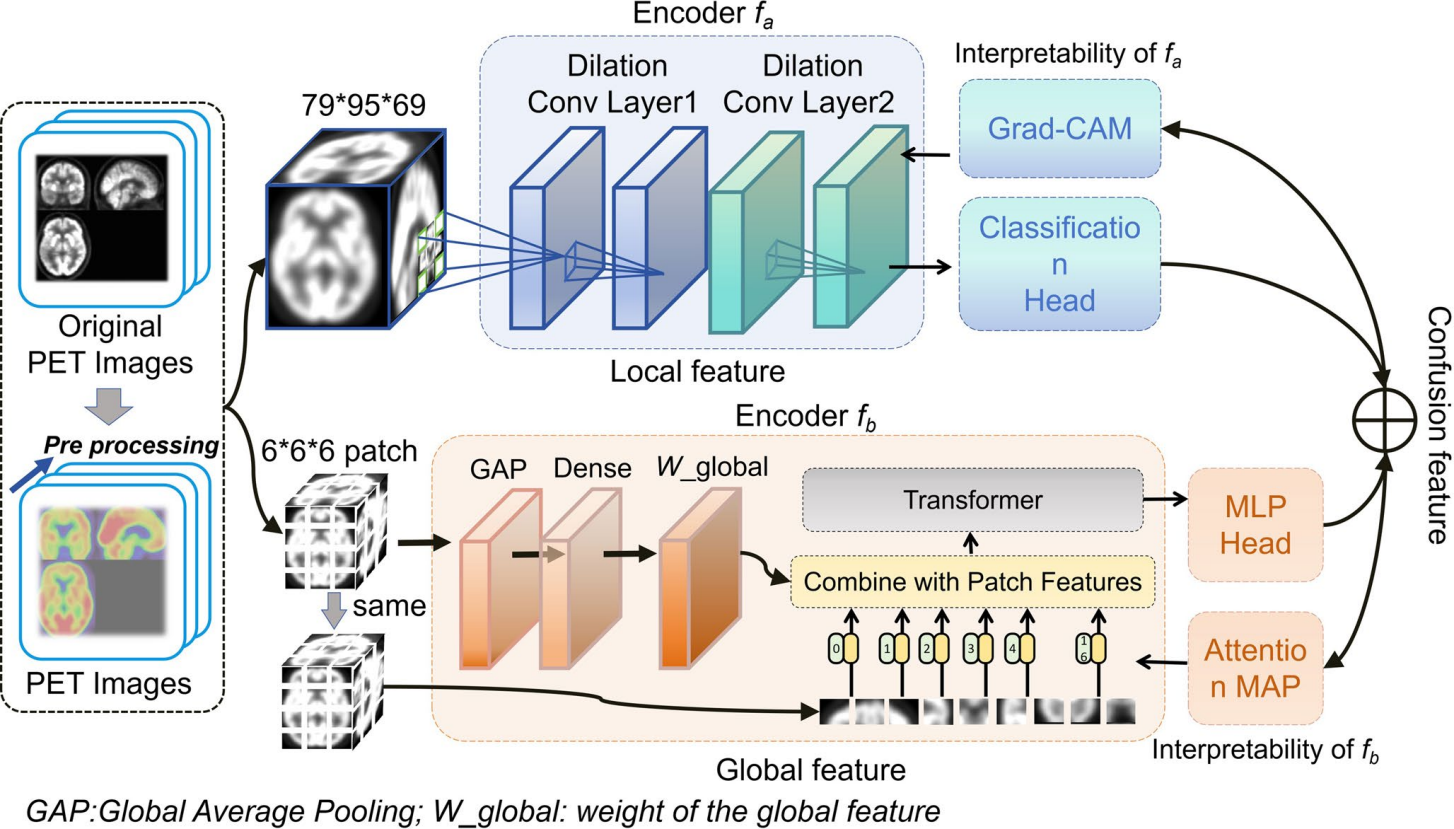
Framework of the RDDNN model

Fig. 3 illustrates the framework of the Dual Deep Neural Network (DDNN), which consists of two independent channels designed to effectively capture both local and global feature information from the input data.

The first channel is based on a DNN that employs dilated convolution modules [35], which are primarily focused on extracting fine-grained local features. This channel processes three-dimensional images with dimensions of 79 × 95 × 69. It includes multiple layers of dilated convolutions that expand the effective receptive field while maintaining spatial resolution, thus enabling the network to gather broader contextual information. The layer configuration consists of a first convolutional layer with 32 filters, a kernel size of 3 × 3 × 3, and a dilation rate of 1; a second convolutional layer with 64 filters, a kernel size of 3 × 3 × 3, and a dilation rate of 2; and a third convolutional layer with 128 filters, a kernel size of 3 × 3 × 3, and a dilation rate of 3. This structured design allows the channel to generate detailed feature maps, facilitating the extraction of intricate local information [36]. In contrast, the second channel utilizes a Transformer framework for global feature extraction. To optimize processing, the input images are resized to 78 × 96 × 66 and divided into overlapping patches using a convolutional kernel of size 6 × 6 × 6. These patches are then processed through a global average pooling layer, which distills global feature representations, enhancing the overall abstraction [37, 38]. The resulting global feature representations are concatenated with the patch vectors obtained through standard Transformer extraction, culminating in a comprehensive feature representation that is fed into the encoder. This integrated approach significantly enhances the network’s capability to capture global information, thereby leveraging both local detail and global context to improve the performance of the DL model.

#### RDDNN model feature selection

To ensure robust and interpretable feature selection, we applied a stepwise filtering strategy comprising three main stages [11]. The feature selection steps are: (1) Features with low variance or inconsistent distributions across cohorts were removed to retain only stable radiomic descriptors (detailed in Supplementary Materials 4 and illustrated in Supplementary Figure S2). (2) We applied Elastic Net-regularized LASSO regression in two phases to prioritize biologically relevant features: (i) an initial screening with α = 0.5 using 10-fold cross-validation and a minimum mean square error (MSE) criterion (λ = 0.21); (ii) a final refinement with α = 1.0 to retain features showing inter-cohort correlation > 0.6. 3) To minimize feature redundancy [12, 14, 39], Pearson correlation analysis (*r* > 0.5) was used to remove highly collinear features. This systematic procedure reduced the initial 2,140 features to 8 stable(The descriptions about spatial localization of selected radiomic features were in Supplementary Figure S3), nonredundant radiomic biomarkers with strong interpretability.

#### RDDNN model classification and comparison

The classification model leveraged the retained features from the DDNN alongside key radiomics features to enhance its predictive capabilities. Through comparative experiments analyzing the results from both the pretraining and training cohorts (detailed in Supplementary Materials 5 and illustrated in Table S4 and S5), we ultimately identified the optimal model for task completion as Extreme Gradient Boosting (XGBoost) [40]. For this model, the parameters were configured to ‘multi: softmax,’ with a learning rate set at 0.025, a Gamma value of 0.1, a maximum depth of 6, and a lambda value of 2. The RDDNN model was compared with various feature models, including pure radiomics features, radiomics features combined with local features, and radiomics features combined with global features. In the three-class classification task, model performance was assessed using AUC, confusion matrices, recall curves, and related metrics (such as AUC score, recall, F1 score, and accuracy) [39].

#### Visual assessment contrast

To reinforce the interpretability and reliability of our proposed approach, we conducted a comparison between the probabilities predicted by the model and the visual assessment made by nuclear medicine specialists from PET images. Nuclear medicine specialists were tasked with assigning confidence scores from visual assessment, reflecting their predicted probabilities, for three specific diseases [41]. In the resulting visual representation, we utilized box plots to illustrate these distributions. The blue box plot represents the confidence score distribution for true-negative cases, while the red box plot represents the distribution for true-positive cases.

### Interpretability and reproducibility of RDDNN model

To enhance the interpretability and reproducibility of the RDDNN model, we employed the SHAP method to quantify the relative contribution of the radiomics-guided dual-channel features in the classifier’s decision-making process across two independent cohorts [42]. As a model-agnostic approach, SHAP computes ‘Shapley values’ that provide a mathematically principled and consistent measure of feature importance. This enables both local and global interpretability by identifying feature-level influences and ranking key contributors [43].

In addition to SHAP, we applied two complementary visualization techniques— Layer-wise Class Activation Mapping (Layer-CAM) and the Rollout Attention Map (RAM) - to investigate the internal attention mechanisms of the local and global channels, respectively [44]. Layer-CAM computes the element-wise product of input gradients and activations across multiple layers to produce fine-grained, layer-specific saliency maps. This allows for voxel-level localization of disease-relevant features within CNN-based local pathways. Conversely, RAM visualizes the attention distribution across token-level representations in the Transformer-based global channel, leveraging self-attention weights to depict long-range contextual dependencies [24, 45]. To facilitate standardized visualization, we used BrainNet Viewer to spatially normalize all 3D attention maps to the MNI template and compute group-level average heatmaps. This enabled consistent anatomical interpretation across subjects and cohorts. Specifically, Layer-CAM maps from all subjects were registered and averaged to reduce inter-subject variability and improve visual clarity. The combination of these two visualization methods improves the interpretability of the model’s decision-making process by revealing spatial attention patterns across local and global channels.

To further complement the above, we included Supplementary Figure S4, which displays layer-by-layer Layer-CAM activation maps for representative IPD, MSA, and PSP cases in both CNN and ViT architectures (detailed in Supplementary Materials 6). These maps reveal channel- and layer-specific activation patterns across the disease subtypes. Additionally, we implemented DeepLIFT-based attribution analysis using the Captum library in PyTorch to further validate the attention behavior of both channels. The results are presented in Supplementary Figure S5.

To assess the stability and reproducibility of the results, we performed pearson correlation analysis to systematically evaluate the cross-center agreement on the importance of SHAP features between two cohorts. Mean absolute SHAP values were used to quantify the linear correlation between cohorts, as shown in Supplementary Figure S6. Furthermore, we used t-distributed stochastic neighbor embedding (t-SNE) to visualize the overall structure of latent representations across both cohorts, as shown in Supplementary Figure S7. In addition, we analyzed the Pearson correlation between the top-ranked RDDNN features and the UPDRS-III scores within each group to assess clinical relevance, as illustrated in Supplementary Figure S8 (detailed in Supplementary Materials 9).

### Statistical analysis

Single-factor analysis of variance (ANOVA) and Bonferroni multiple comparisons were used to compare continuous variable information, while chi-square tests assessed gender information. All statistical analyses were conducted using SPSS version 24.0. A two-tailed p-value of less than 0.05 was considered statistically significant.

## Results

### Subjects

The clinical and demographic information of the patients is presented in Table 1. Notably, patients with PSP were statistically significantly older than those in the other groups (*p* < 0.001). Additionally, significant differences in motor symptom assessment were observed among patients with Parkinsonism (*p* < 0.001). Specifically, patients diagnosed with IPD exhibited lower scores on the Unified Parkinson’s Disease Rating Scale III (UPDRS III), indicating less severe motor impairment and disability compared to other Parkinsonism.

**Table 1:** The clinical characteristics of all cohorts

Clinical parameters	Huashan loansharking PET imaging data set (Chinese cohort)							Additional Independent test cohort (German cohort)
	Pretraining cohort	Training cohort			Blind-test cohort			
		Overall	Short symptom duration (≤ 2 y)	Long symptom duration (> 2 y)	Overall	Baseline	Follow-up	
IPD								
Patient (*n*)	241	299	136	163	211	66	66	34
Sex (male/female)	154/87	166/133	73/63	93/70	130/81	43/23	43/23	21/13
Age at PET (y)	50.0 ± 15.5	60.2 ± 8.5	59.1 ± 9.0	61.0 ± 8.0	60.0 ± 7.6	60.0 ± 7.9	62.1 ± 7.9	72.9 ± 9.5
Symptom duration at PET (mo)	–	45.3 ± 46.0	13.0 ± 5.9	72.3 ± 47.4	39.0 ± 41.3	26.0 ± 24.1	53.4 ± 24.2	44.5 ± 32.9 (18/34)
UPDRS III	–	27.0 ± 14.3	18.9 ± 8.9	33.8 ± 14.5	22.8 ± 12.1	19.6 ± 9.1	24.2 ± 10.1	12.0 ± 3.6 (3/34)
Clinical follow-up (mo)	–	–	–	–	46.8 ± 30.4	–	64.5 ± 25.3	19.1 ± 21.8 (14/34)
MSA*								
Patient (n) (MSA-C/MSA-P)	79	150 (57/93)	90 (39/51)	60 (18/42)	61 (21/40)	22 (8/14)	22 (8/14)	17 (8/8/1)
Sex (male/female)	42/37	78/72	47/43	31/29	32/29	14/8	14/8	10/7
Age at PET (y)	57.5 ± 10.6	57.8 ± 8.0	56.5 ± 8.1	59.6 ± 7.4	58.5 ± 6.3	58.3 ± 7.4	60.3 ± 7.3	61.3 ± 8.3 (17/17)
Symptom duration at PET (mo)	–	24.3 ± 17.1	13.9 ± 6.0	39.9 ± 16.5	27.0 ± 20.1	22.1 ± 11.8	45.6 ± 12.5	30.0 ± 22.2 (17/17)
UPDRS III	–	30.6 ± 14.5	25.9 ± 12.4	37.6 ± 14.7	29.3 ± 14.4	23.5 ± 8.2	36.4 ± 11.1	34.6 ± 12.8 (11/17)
Clinical follow-up (mo)	–	–	–	–	30.7 ± 18.2	–	41.7 ± 16.4	22.6 ± 22.4 (17/17)
PSP								
Patient (n)	78	98	34	64	58	20	20	39
Sex (male/female)	45/33	60/38	23/11	37/27	39/19	17/3	17/3	21/18
Age at PET (y)	64.6 ± 8.6	67.2 ± 8.0	65.0 ± 9.3	68.5 ± 6.9	65.1 ± 6.6	64.8 ± 7.5	67.0 ± 7.2	70.0 ± 7.1
Symptom duration at PET (mo)	–	35.0 ± 20.7	15.3 ± 5.4	45.5 ± 18.0	34.1 ± 22.7	32.4 ± 22.0	58.8 ± 22.8	22.4 ± 15.7 (37/39)
UPDRS III	–	30.1 ± 13.5	28.0 ± 11.0	31.2 ± 14.6	26.8 ± 11.0	23.0 ± 10.4	34.6 ± 15.9	37.0 ± 15.9 (20/39)
Clinical follow-up (mo)	–	–	–	–	25.1 ± 15.7	–	37.5 ± 12.9	22.2 ± 13.8 (17/39)
HC								
Patient (n)	223	–	–	–	–	–	–	–
Sex (male/female)	104/119	–	–	–	–	–	–	–
Age at PET (y)	58.9 ± 10.5	–	–	–	–	–	–	–
UPDRS III	–	–	–	–	–	–	–	–

*mo* month

Diagnosis information: Table  S1

Data are shown as mean ± SD. In German cohort, associated numbers of subjects with these items are provided together with statistics information (subject number with certain item/total subject number)

*UPDRS III *Unified Parkinson’s Disease Rating Scale III; *MSA-C/MSA-P *MSA-cerebellar/MSA-parkinsonian

In the pretraining cohort, a statistically significant gender imbalance was also present (*p* < 0.001), with more females in the HCs group and more males in the patient groups. This information provides a critical context for understanding patient characteristics and their potential implications on the study’s findings.

### Selected fusion features

**Fig. 4:**
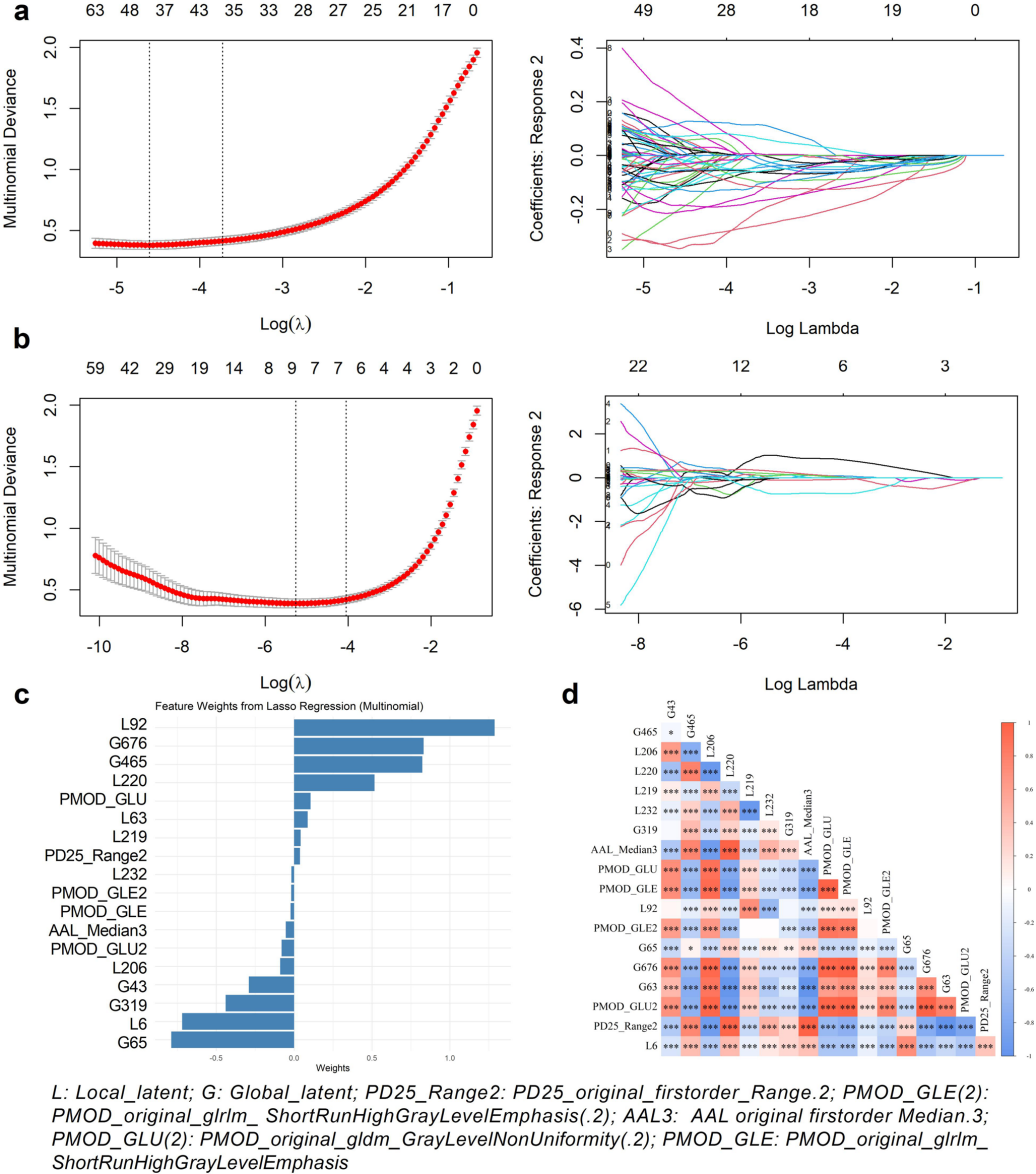
The RDDNN feature screening analysis procedure involves multiple stages. (a) Lasso coefficient profiles are examined across various logarithmic values (λ) to identify the optimal regularization parameter (λ) for the LASSO model using the minimum criteria. (b) This evaluation continues by refining the selection of adjustment parameters (λ) based on the Lasso coefficient profiles at different logarithmic values (λ) and minimum criteria. (c) The feature weights of the selected features by LASSO are assessed. (d) A heat map of characteristics is generated based on the selected features’ results of the Pearson correlation coefficient

As shown in Fig. 4, the final fusion set included 8 stable radiomics features and 10 deep learning-derived latent features (5 local and 5 global). The LASSO weight plots (Fig. 4c) demonstrate the contribution of each retained feature, while the Pearson correlation heatmap (Fig. 4d) confirms low redundancy. To further illustrate the anatomical relevance of the radiomic features, we visualized their spatial locations using BrainNet Viewer software (Supplementary Figure S3). The retained features were localized to clinically meaningful regions, including the bilateral superior frontal gyri (medial and orbital), supplementary motor areas (SMA), the striatum (right putamen, caudate nucleus, and pallidum), as well as the middle and inferior occipital gyri. These regions are strongly implicated in the pathophysiological processes of Parkinsonian syndromes, affirming both the statistical and biological validity of the selected feature set.

### Classification results

**Fig. 5:**
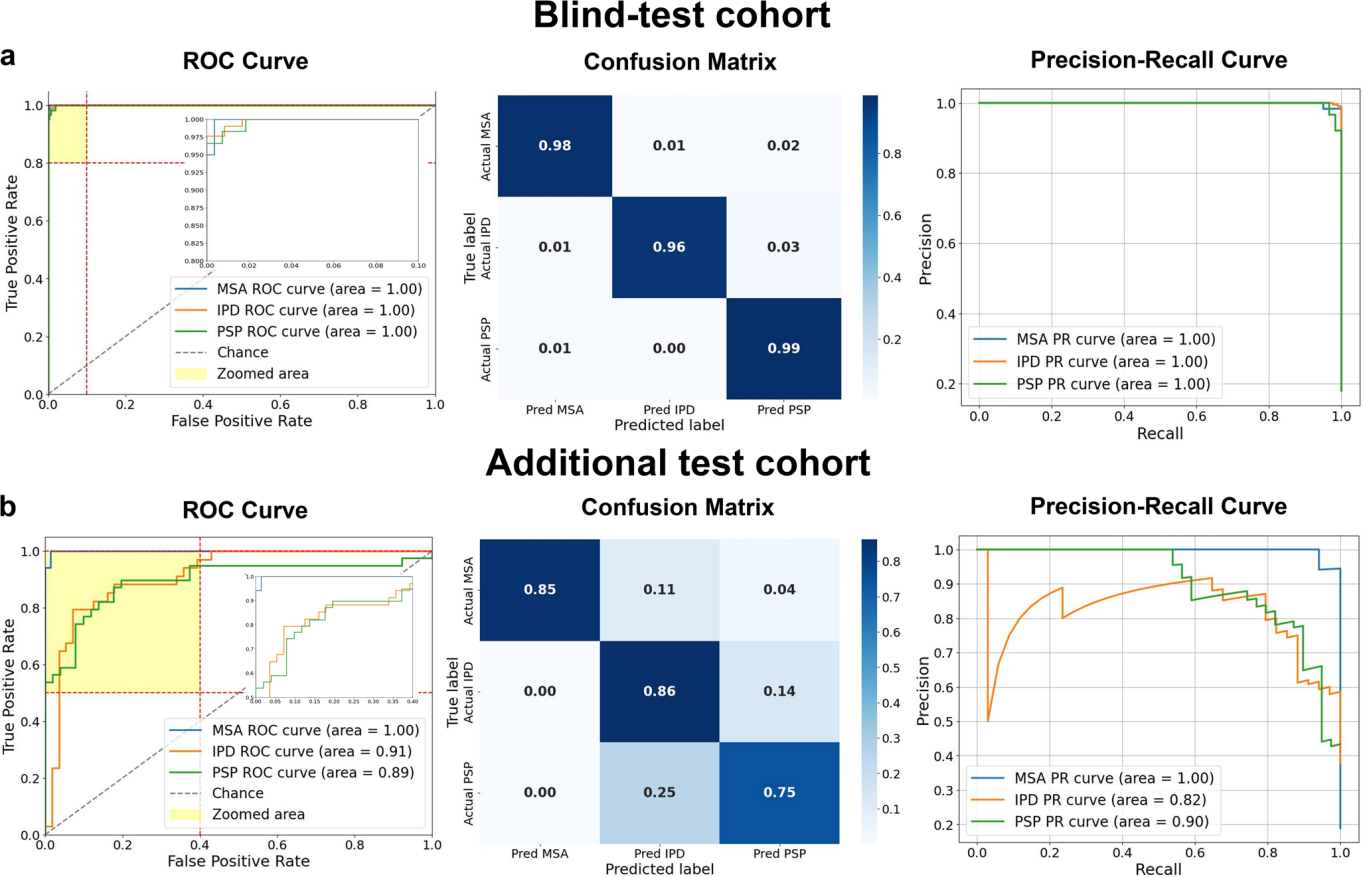
ROC curves, confusion matrices, and recall curves for two independent test cohorts. (a) Results for the blind-test cohort. (b) Results for the additional test cohort. Each panel presents the ROC curve, which illustrates the model’s sensitivity and specificity; the confusion matrix, detailing the counts of true positives, true negatives, false positives, and false negatives; and the recall curve, which demonstrates the model’s recall performance across various thresholds

As shown in Fig. 5, the RDDNN model achieved excellent multi-class performance in both cohorts. In the blind-test set, all three classes reached an AUC of 0.99, with near-perfect separation in the ROC and PR curves. The confusion matrix indicated minimal misclassification across MSA, IPD, and PSP. In the external test set, classification performance remained strong, with AUCs of 0.91 (IPD) and 0.90 (PSP), and MSA achieving the highest precision. These results confirm the model’s reliable generalization across distinct populations.

In the blind-test cohort, the RDDNN model achieved an impressive accuracy of 0.976, an AUC of 0.992, an F1 score of 0.979, a recall of 0.991, and precision of 0.969. These results reflect the model’s superior diagnostic ability in identifying the condition accurately. In the additional test cohort, the RDDNN model maintained strong performance with an accuracy of 0.814, an AUC of 0.941, an F1 score of 0.830, a recall of 0.817, and precision of 0.856. The specific performance metrics for both cohorts are presented in Table 2, which provides a comprehensive overview of the RDDNN model’s diagnostic capabilities compared to traditional methods, further highlighting the advantages of the proposed approach.

**Table 2:** Three-classification performance of each method

Cohort	Method	Accuracy (%)	AUC score (%)	F1 score (%)	Recall (%)	Precision (%)
Blind-test Cohort	radiomics	0.909	0.952	0.877	0.883	0.873
	radiomics with local	0.951	0.981	0.929	0.935	0.922
	radiomics with globle	0.956	0.988	0.951	0.967	0.936
	**RDDNN**	**0.976**	**0.992**	**0.979**	**0.991**	**0.969**
Additional test Cohort	radiomics	0.712	0.842	0.728	0.717	0.786
	radiomics with local	0.734	0.844	0.739	0.744	0.750
	radiomics with globle	0.798	0.928	0.824	0.832	0.841
	**RDDNN**	**0.814**	**0.941**	**0.830**	**0.817**	**0.856**

### Visual assessment contrast

**Fig. 6:**
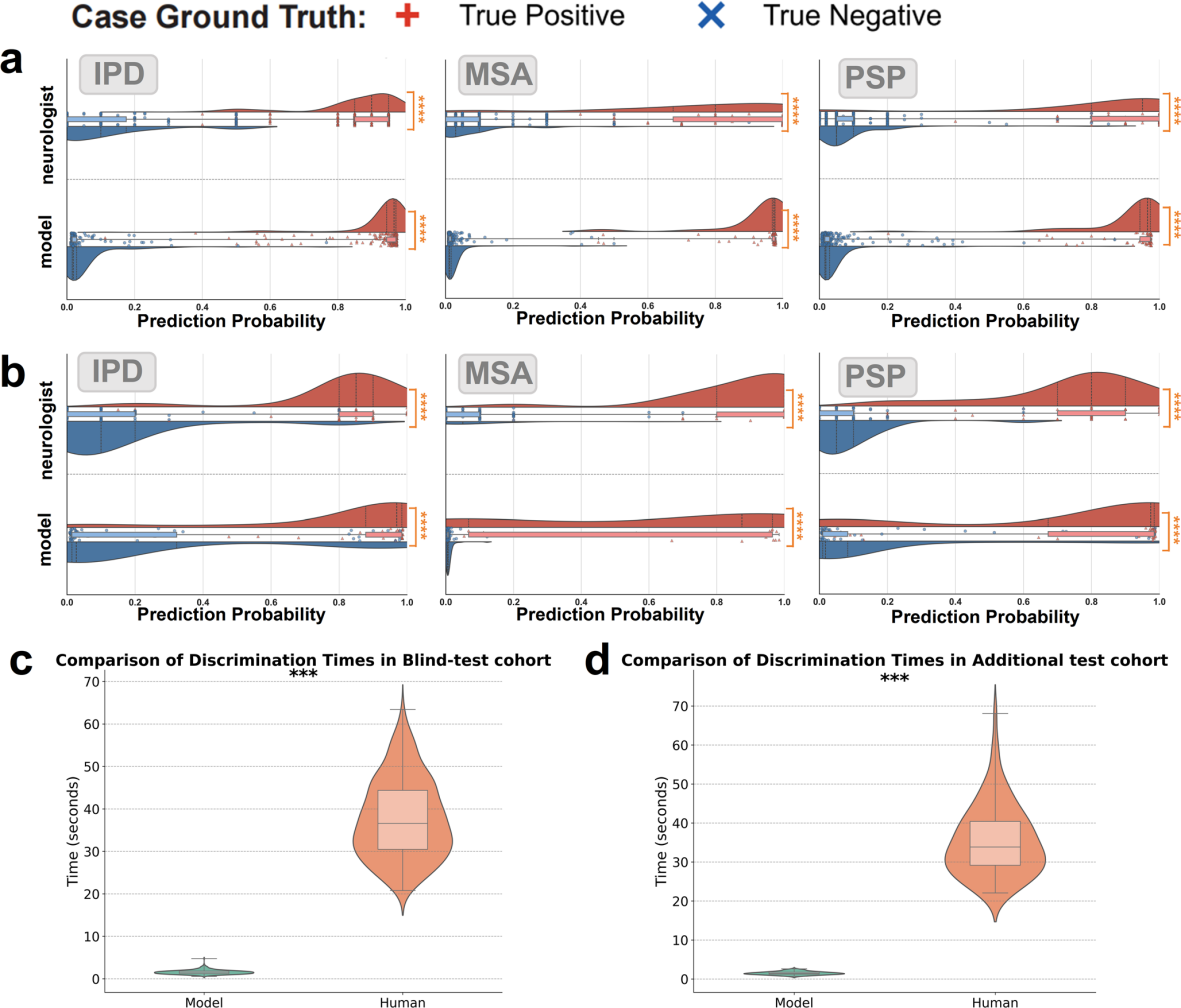
Head-to-head comparison between model and neurologist. Probability scores predicted by the model were shown with assessments provided by practicing clinicians. (**a**) Comparison of the assessment of IPD, MSA and PSP diseases in blind-test cohort. (**b**) Assessment and comparison of IPD, MSA and PSP diseases in additional test cohort. (**c**) Time efficiency of models and nuclear medicine specialists in blind-test cohort (d) Time efficiency of models and nuclear medicine specialists in additional test cohort. In the visual representation, the blue box plot represents the distribution of true negative confidence scores, while the red box plot represents true positive cases. The symbol “+” represents the true case and “x” represents the true negative case. Significance When *p* ≥ 0.05, ns (not significant) was used to indicate the level. * is *p* < 0.05; ** is *p* < 0.01; **** is *p* < 0.0001; These levels were pairwise compared using an unadjusted Mann-Whitney U test

The evaluation results comparing the performance of the model and nuclear medicine specialists are illustrated in Fig. 6. Utilizing the Mann-Whitney U test for analysis, we found that both the model and nuclear medicine specialists successfully distinguished true negative cases from true positive cases, with a statistically significant result (*p* < 0.001). In Fig. 6a, it is evident that the assessment probability of the model exhibits greater stability compared to that of the nuclear medicine specialists when evaluations were conducted at the same center. However, Fig. 6b highlights a limitation of the model, showing that it was less effective in distinguishing MSA diseases compared to the nuclear medicine specialists in the additional test cohort. As shown in Fig. 6c and d, the model significantly reduced evaluation time compared to nuclear medicine specialists across both cohorts (*p* < 0.001), achieving a reduction of over 80%. This highlights its practical utility in time-sensitive, high-throughput clinical settings.

### Interpretability and reproducibility assessment

**Fig. 7:**
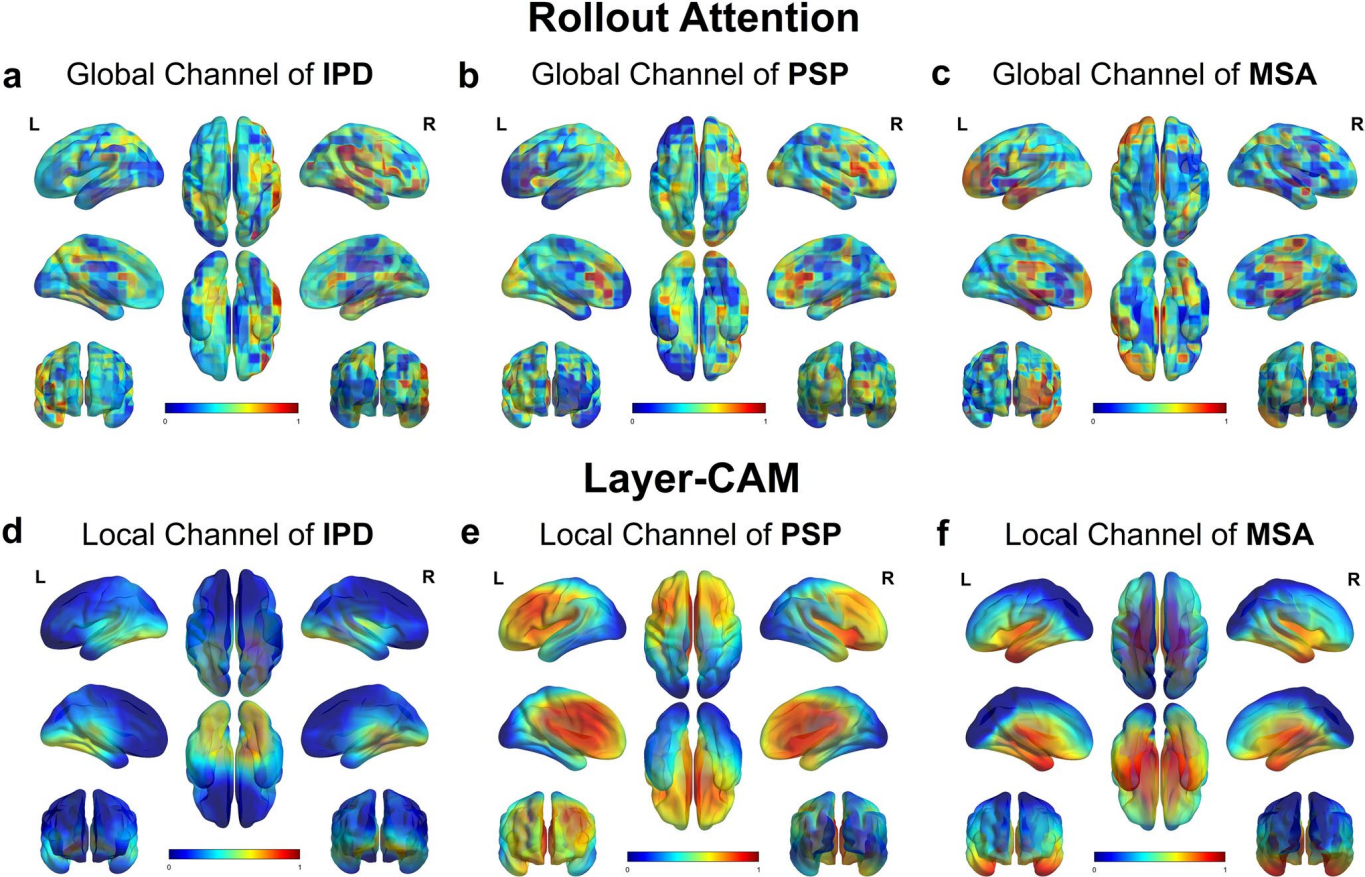
(**a-c**) Global channel attention maps for IPD, PSP, and MSA, respectively, derived from Transformer-based architectures (RAM). (**d-f**) Local channel activation maps for IPD, PSP, and MSA, respectively, derived from CNN-based architectures (Layer-CAM)

As shown in Fig. 7, the local channel attention (Fig. 7d-f) derived from the final Layer-CAM layer reveals consistent focus on midbrain, putamen, and caudate nucleus—particularly in PSP and MSA groups. These activations align well with established metabolic deficits reported in prior PET literature [41, 46–48]. In contrast, global channel attention (Fig. 7a-c) demonstrates broader cortical coverage, including frontal and parietal lobes, while still capturing signals in core disease-relevant areas such as the thalamus and basal ganglia. Taken together, these results demonstrate complementary attention patterns across anatomically plausible regions, reflecting the distinct but synergistic spatial reasoning mechanisms embedded in the dual-channel RDDNN architecture.

**Fig. 8:**
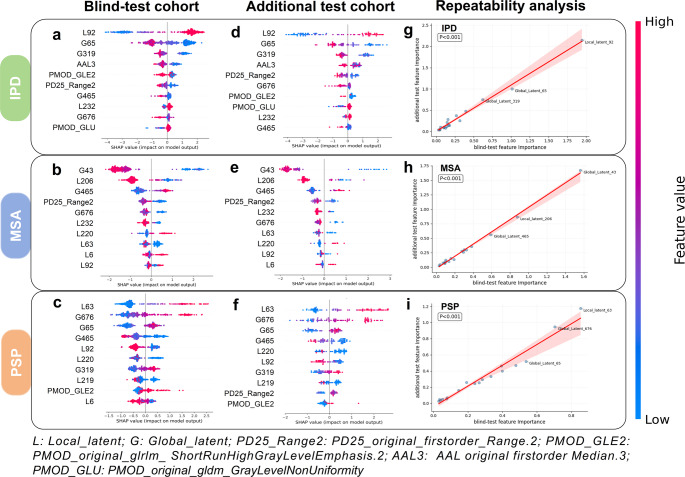
SHAP Results for the XGBoost Classifier in IPD, MSA, and PSP Classification. (**a**) the effect of features on the IPD classification decision in the blind-test cohort. (**b**) the effect of features on the MSA classification decision in the blind-test cohort. (**c**) the effect of features on the PSP classification the effect. (**d**) the effect of features on the IPD classification decision in the additional test cohort. (**e**) the effect of features on the MSA classification decision in the additional test cohort. (**f**) the effect of features on the PSP classification in the additional test cohort. (**g**) Correlation of SHAP values reflect feature importance for IPD subtype classification between two cohorts. (**h**) Correlation of SHAP values reflect feature importance for MSA subtype classification between two cohorts. (**i**) Correlation of SHAP values reflect feature importance for PSP subtype classification between two cohorts

Fig. 8 summarizes the results of the SHAP analysis conducted on both cohorts, which were integrated with the XGBoost classifier to differentiate between IPD, MSA, and PSP subtypes. Notably, features such as Global_latent_43, Local_latent_92, and Local_latent_63 demonstrated substantial classification contributions in both the blind-test and additional test cohorts, providing optimal discrimination for MSA, IPD, and PSP classifications, respectively. Additionally, Local_latent_37 and Local_latent_109, along with two key radiomics features—PD25_original_firstorder_Range.2 and PMOD_original_glrlm_ShortRunHighGrayLevelEmphasis.2—played essential roles in distinguishing these categories across diverse populations. In Figs. 8g, h and i, the strong concordance of SHAP feature importance rankings between two cohorts (Pearson’s *r* = 0.98–0.99, *p* < 0.001) establishes the model’s invariant dependence on key biomarkers across heterogeneous datasets. The consistency of contributing features across different populations further emphasizes the reproducibility and applicability of the model.

## Discussion

In this study, we validated the clinical classification performance of the RDDNN model using FDG-PET imaging data from two centers. Our findings demonstrate that the RDDNN framework effectively addresses core diagnostic challenges in this context. To ensure robust generalizability, a structured three-step feature selection strategy—comprising feature stability validation, LASSO refinement, and cross-channel redundancy control—was employed to ensure feature robustness across different cohorts. Our analysis revealed that RDDNN features identified common metabolic characteristics across different ethnic groups and various networks. By fusing dual-channel features, we were able to enhance classification accuracy while ensuring the interpretability of each channel remained intact. Notably, the inclusion of bi-centric validation allowed us to evaluate model performance across diverse cohorts, specifically among Chinese and German populations, thus demonstrating the reproducibility of our model.

Previous studies have demonstrated a significant overlap in the clinical manifestations of IPD, MSA, and PSP, leading to a misdiagnosis rate ranging from 20 to 30% [49]. Such scenarios can result in delays in clinical treatment and adversely affect the prognosis of each condition [50]. Several recent studies have explored the application of deep learning in differentiating Parkinsonian syndromes. Wu et al. [13]. employed FDG-PET–based deep metabolic imaging indices with a 3D convolutional neural network to classify IPD, MSA, and PSP, achieving high sensitivities ranging from 84.5 to 98.1% and specificities up to 99.2% in the blind test cohort. Ling et al. [18]. proposed a radiomics-guided DenseNet model that reached sensitivities of 95.7% for IPD, 90.1% for MSA, and 91.2% for PSP, highlighting the role of handcrafted radiomics features in enhancing interpretability. Zhao et al. [24]. applied a 3D CNN to dopamine transporter PET images, obtaining diagnostic sensitivities of 90.7% for IPD, 84.1% for MSA, and 78.6% for PSP. Compared with these approaches, the RDDNN model achieving an accuracy of 98% in the blind-test cohort and 81% in the additional test cohort, thereby showcasing its good reproducibility. Our ablation comparisons indicate that the model achieves optimal performance under multi-scale fusion. The superior performance of the RDDNN model is underpinned by several key design advantages. First, the model integrates dilated convolutional networks for fine-grained local feature extraction with Transformer-based self-attention mechanisms to capture broad contextual information. This dual-channel architecture allows the network to learn multiscale metabolic signatures, enhancing its ability to localize disease-relevant regions and represent complex spatial heterogeneity. Second, the separation of local and global pathways supports complementary encoding of region-specific abnormalities and global metabolic disruptions, which is particularly valuable in disorders like APS with diffuse and variable pathological involvement. Finally, by fusing radiomics features with deep learning embeddings, the model benefits from a hybrid representation that balances interpretability and discriminative power, enabling consistent classification performance across cohorts with distinct population characteristics and disease trajectories.

Importantly, in this study, Layer-CAM visualizations revealed that the regions receiving high activation in the classification process—such as the red nucleus, raphe nucleus, midbrain, nucleus accumbens, and cingulate gyrus [51, 52]—spatially correspond to brain areas previously reported as metabolically impaired in atypical Parkinsonian syndromes [53]. Rather than directly identifying hypometabolism, the model implicitly learned to focus on clinically informative regions with high discriminative value across different subtypes. These findings align with prior FDG-PET studies, which observed reduced metabolism in the putamen, pons, and cerebellum in MSA patients, and in the caudate nucleus, thalamus, midbrain, and cingulate gyrus in PSP patients, distinguishing them from IPD [21, 50, 54]. The convergence between model-driven attention and known pathological sites enhances the clinical plausibility of the RDDNN framework. By integrating dual-channel feature learning and radiomics-guided supervision, RDDNN facilitates the extraction of semantically meaningful and spatially localized patterns across multiple brain regions, extending beyond pre-defined ROI boundaries. This provides a richer pathological representation than conventional radiomics pipelines limited by manual segmentation and fixed feature scopes. Additionally, results of the SHAP feature importance analysis demonstrated that the RDDNN model exhibited highly consistent feature contributions across the bi-centric cohort analyses. SHAP values provide critical insights into how each feature influences model predictions, which is essential for enhancing the interpretability and reproducibility of predictive models in clinical settings. Among radiomic features, GLRLM-based metrics emerged as highly ranked contributors, reflecting the localized aggregation of high gray-level intensities—an interpretable and physiologically meaningful trait in PET imaging [11]. While deep learning features such as Local_latent_92 remain abstract in numerical form, their selection and consistent contribution—combined with anatomical visualization via attention maps—enhance the interpretability of the entire framework. Rather than positioning radiomics and deep learning as mutually exclusive, RDDNN integrates both to enable multilevel interpretability: mathematical traceability from radiomics, and spatial rationale from DL channel visualizations.

Notably, Layer-CAM and RAM derived from our dual-channel architecture do not always spatially overlap, which is an expected and reasonable outcome given their distinctive roles. Layer-CAM heatmaps primarily reflect regions of local relevance, correlating with focal neuroanatomical changes, whereas attention-based maps derived from the Transformer module capture broader, global dependencies and cross-regional interactions. This divergence reinforces the complementary nature of the two interpretability strategies. For clinical end-users, we recommend that radiologists and nuclear medicine specialists leverage both types of explanations. Layer-CAM can aid in pinpointing critical localized abnormalities, while Rollout Attention Maps can provide insights into more distributed or complex disease patterns. By integrating both perspectives, clinicians are empowered to make more informed and transparent decisions, especially in cases involving atypical or diffuse pathologies.

In addition, we compared the predictive performance of our model with the visual assessments conducted by nuclear medicine specialists. The results indicated that while both the model and the nuclear medicine specialists effectively distinguished between subtypes within the groups (*p* < 0.001), the model demonstrated predictive performance that was comparable to, or even superior to, that of the nuclear medicine specialists in differentiating between various disease subtypes. Furthermore, in terms of time efficiency, it was evident that the model required significantly less time to make predictions compared to the nuclear medicine specialists (*p* < 0.001). These findings suggest that our proposed model can effectively assist clinicians in managing the challenges associated with a high volume of imaging data and the prolonged interpretation times, thereby demonstrating its potential as a valuable clinical decision support tool.

Finally, we must acknowledge the limitations of our study. Firstly, due to the limited number of training samples, we did not employ more complex DL networks, which typically require larger datasets for effective model convergence. Other DL architectures may offer varying diagnostic capabilities by extracting information from FDG-PET images across different dimensions, but this would necessitate further validation. Additionally, our validation of this framework was restricted to FDG-PET images; other specific techniques, such as dopaminergic imaging [55], could potentially offer greater predictive capabilities. We plan to validate our model using other imaging biomarkers for Parkinsonism, including dopaminergic PET imaging [56]. The clinical utility of the model across different imaging modalities requires further investigation to determine its effectiveness and versatility in various clinical settings. Thirdly, it is important to note that the present study focused on integrating and systematically evaluating mainstream methods, such as radiomics, CNNs, Transformers, and established interpretability tools, rather than proposing a fundamentally novel or inherently interpretable model architecture. In the future, advancing toward models with intrinsic transparency and interpretability from the input stage—rather than relying solely on post hoc explanations—as well as exploring more advanced interpretable machine learning approaches, will be a major direction for research in this field.

## Conclusion

Currently, the collaboration between AI and human intelligence has become essential for understanding complex diseases and making accurate diagnoses to provide optimal treatment. In this comprehensive bi-centric study, we introduced an innovative classification modeling approach based on RDDNN using FDG-PET images. Our findings demonstrate the exceptional predictive capability of RDDNN features in accurately differentiating between forms of Parkinsonism, underscoring their robustness and reliability in aiding disease stratification and management. The RDDNN model can be interpreted with consistency in anatomical locations, as well as through anatomical and physiological explanations. These preliminary assessments highlight the potential of the RDDNN approach as a valuable clinical tool for enhancing supportive clinical decision-making.

## Supplementary Information

Below is the link to the electronic supplementary material.

Supplementary Material 1

## Data Availability

The datasets generated during and/or analysed during the current study are available from the corresponding author on reasonable request.
