# Supplementary material for: Enhancing interpretability of AI with radiomics-based deep neural network: proof of concept in the classification of Parkinsonian syndromes with 18F-FDG PET imaging
Source: Eur J Nucl Med Mol Imaging. 2025 Sep 11;53(3):1962–79. doi: 10.1007/s00259-025-07478-7 (PMC12860829; doi:10.1007/s00259-025-07478-7)
Supplement: Supplementary file 1 — Supplementary Material 1 [file 259_2025_7478_MOESM1_ESM.docx]

**Enhancing Interpretability of AI with Radiomics-based Deep Neural Network: Proof of Concept in the Classification of Parkinsonian Syndromes with ^18^F-FDG PET Imaging**

**Tipical case of the SUVR MAP about IPD/MSA/PSP/HC in groups**

**
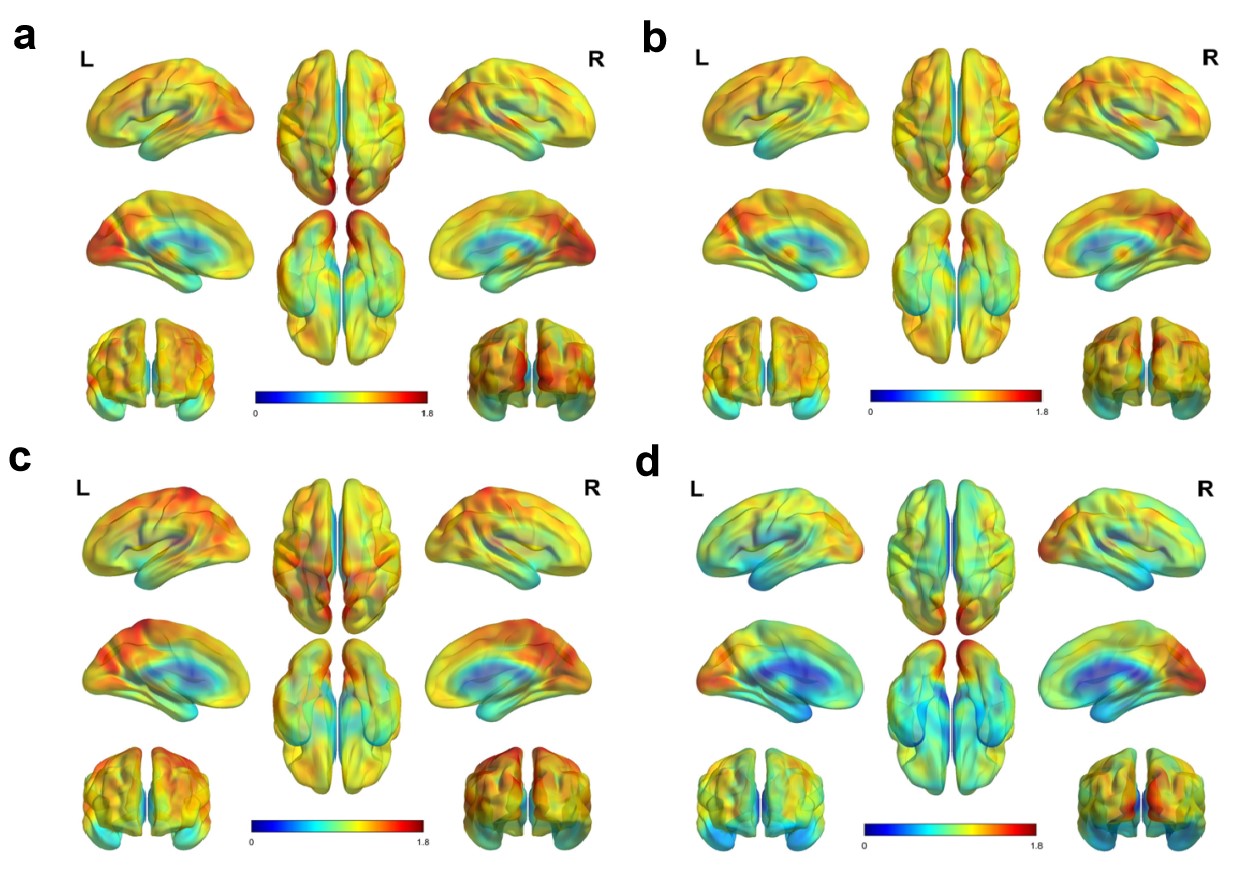
**

**FigureS1:** Spatial Distribution of Standardized Uptake Value Ratio in Parkinsonism. (a) MSA Case: Female, 60 years old, symptom duration = 12 months. (b) IPD Case: Female, 58years old, symptom duration = 13 months. (c) PSP Case:Female, 61 years old, symptom duration = 12 months. (d) Hormal Control: Female, 59 years old. All standardized uptakevalue ratio maps were computed using the global mean brainuptake as the reference region. Warmer colors indicate regions with higher relative glucose metabolism, while cooler colors represent areas of lower metabolic activity.

**Supplementary Materials 1: Detailed Information about the Chinese cohort**

In the Chinese cohort, a total of 1,275 parkinsonian patients were enrolled. These patients were categorized into a pre-training cohort, training cohort, and blind-test cohort based on the certainty of their clinical diagnosis and the availability of follow-up clinical data (at least one year after PET imaging).

Pre-training cohort (241 IPD, 79 MSA, 78 PSP): Comprised patients with clinically possible diagnoses of IPD, MSA, or PSP, used for preliminary training of the PDD-Net. To align with the study's objective of deriving deep metabolic imaging (DMI) indices for differential diagnosis—and acknowledging that MSA/PSP diagnostic criteria specify onset age thresholds—all patients with disease onset younger than 40 years were assigned to this cohort. Additionally, patients with definite clinical diagnoses but lacking detailed medical records were included.

Training cohort (299 IPD, 150 MSA, 98 PSP): Included patients with clinically definite diagnoses confirmed by follow-up evaluations but without formal long-term clinical follow-up. This cohort was utilized for fine-tuning the PDD-Net and cross-validation to extract DMI indices. Patients were further divided into subgroups based on symptom duration: short (≤2 years) and long (>2 years).

Blind-test cohort (211 IPD, 61 MSA, 58 PSP): Composed of patients with clinically confirmative diagnoses established through at least one formal clinical follow-up exceeding one year after PET imaging, used for independent testing of DMI indices. Algorithm developers were blinded to the clinical details and diagnoses of individuals in this cohort. A subset of 108 patients underwent repeat PET scans at follow-up in addition to baseline (first diagnosis) imaging. For analytical purposes, FDG-PET images were labeled as:

- "Overall": Baseline images of all 330 blind-test patients

- "Baseline": Baseline images of the 108 repeat-scanned patients

- "Follow-up": Follow-up images of the 108 repeat-scanned patients

Clinical diagnoses were based on the most recently published criteria[1-3]. Diagnoses of IPD- and PSP-initially made using older criteria[4,5] in the training and blind-test cohorts were reconfirmed via chart review or follow-up using the latest criteria[1,2]. Detailed diagnostic distributions by criteria are listed in Table S1.

Table S1 .The detailed information of the clinical diagnosis according to different versions of diagnostic criteria.

|  | **Clinical** **Criteria** | **Pre-training** **Cohort** | **Training** **Cohort** | **Blind-test** **Cohort** |
| --- | --- | --- | --- | --- |
| IPD | New^[2,5]^ | 112 | 185 | 77 |
|  | Old^[5]^ | 129 | 114 | 134 |
| PSP | New^[4]^ | 36 | 66 | 29 |
|  | Old^[4]^ | 42 | 32 | 29 |

*PSP consists of 165 PSP-Richardson syndrome (PSP-RS) and 69 other subtypes

Note: All patients diagnosed with old criteria were reconfirmed with the new diagnosis criteria.

**Supplementary Materials 2: Data difference between Chinese and German cohort**

PET/CT protocol difference between Chinese and German cohort

Chinese Cohort

After attenuation correction performed using low-dose CT, the emission scan was acquired at 60-minute post injection of approximately 185 MBq ^18^F-FDG and lasted 10 minutes (Siemens Biograph 64 HD PET/CT, Siemens, Germany). PET images were reconstructed by using the ordered subset expectation maximization method following corrections for scatter, dead time, and random coincidence.

German Cohort

(1) Siemens ECAT EXACT HR+ and GE Discovery 690

FDG-PET images were acquired on a GE Discovery 690 PET/CT scanner or a Siemens ECAT EXACT HR+ PET scanner.

All patients had fasted for at least six hours and had a maximum plasma glucose level of 150 mg/dl at time of scanning. A single intravenous dose of 140 ± 7 MBq FDG was administered while the patients rested in a room with dimmed light and low noise level, where they remained undisturbed for 20 minutes. After positioning in the scanner, a series of three static emission frames of five minutes each was acquired from 30 to 45 min p.i. on the GE Discovery 690 PET/CT, or from 30 to 60 min p.i. on the Siemens ECAT EXACT HR+ tomograph. A low-dose CT scan or a transmission scan with external 68Ge-source performed just prior to the static acquisition was used for attenuation correction. PET data were reconstructed iteratively (GE Discovery 690 PET/CT) or with filtered-back-projection (Siemens ECAT EXACT HR+ PET). After correction for movement between frames, the static scans were averaged.

(2) Siemens Biograph 64

The PET data were acquired on a Siemens Biograph True point 64 PET/CT (Siemens, Erlangen, Germany). The dynamic brain PET data were acquired in 3-dimensional list-mode over 20min and reconstructed into a 336x336x109 matrix (voxel size: 1.02×1.02×2.03 mm3) using the built-in ordered subset expectation maximization (OSEM) algorithm with 4 iterations, 21 subsets and a 5mm Gaussian filter. A low dose CT served for attenuation correction.

Table S2 The comparison of the PET/CT protocols between Chinese and German cohorts

|  | Chinese cohort | German cohort | | |
| --- | --- | --- | --- | --- |
|  | Siemens Biograph64 | Siemens ECAT  Exact HR+ | GE Discovery 690 | Siemens Biograph  64 |
| Sensitivity | 4.5 kcps/MBq | 6.65 kcps/MBq | 7.5 cps/kBq | 4.5 kcps/MBq |
| Transverse Resolution | 4.2± 0.3 mm | 4.39 mm | 4.70 | 4.2± 0.3 mm |
| AxialResolution | 4.5± 0.3 mm | 5.10 mm | 5.06 | 4.5± 0.3 mm |
| Peak NEC | 93 kcps | 37 kcps | 139.1 kcps | 93 kcps |
| Scatter Fraction | 32% | 46.9% | 37% | 32% |
| Injection dose (MBq) | ~185 | 140 ± 7 | 140 ± 7 | / |
| Acquisition time p. i.(min) | 60 | 95 | 30 | 30 |
| Imaging duration (min) | 10 | 20 | 15 | 20 |
| Reconstruction method | OSEM | IFBP | Iterative | OSEM |
| Attenuation correction | CT | 68 Ge transmission | CT | CT |
| Reconstructed voxelsize | 2.03×2.03×1.5 mm3 | 1.4×1.4×2.4 mm3 | / | 1.02×1.02×2.03  mm3 |
| Smooth | Gaussian 10mm | / | / | Gaussian 5mm |
| Eye mask | yes | / | / | / |
| Fasting | >6 hour | >6 hour | >6 hour | >6 hour |
| Blood glucose level | <150 mg/dl | <150 mg/dl | <150 mg/dl | <150 mg/dl |

**Supplementary Materials 3: descriptions about all 107 radiomics features**

Table S3. The descriptions about all 107 radiomics features

| **Feature category** | **Label** | **Feature name** |
| --- | --- | --- |
| Shape feature | 1  2  3  4  5  6  7  8  9  10  11  12  13  14 | Elongation  Flatness  LeastAxisLength MajorAxisLength  Maximum2DDiameterColumn Maximum2DDiameterRow  Maximum2DDiameterSlice Maximum3DDiameter  MeshVolume  MinorAxisLength Sphericity  SurfaceArea  SurfaceVolumeRatio VoxelVolume |
| First order features | 15  16  17  18  19  20  21  22  23  24  25  26  27  28  29  30  31  32 | 10Percentil90Percentile Energy  Entropy  InterquartileRange Kurtosis  Maximum  MeanAbsoluteDeviation  Mean  Median  Minimum Range  RobustMeanAbsoluteDeviation  RootMeanSquared  Skewness  TotalEnergy Uniformity Variance |
| Gray Level Cooccurrence  Matrix(GLCM) Features | 33  34  35  36  37  38  39  40  41  42  43  44  45  46  47  48  49  50  51  52  53  54  55  56 | Autocorrelation  ClusterProminence  ClusterShade  ClusterTendency  Contrast  Correlation  DifferenceAverage  DifferenceEntropy  DifferenceVariance  Id  Idm  Idmn  Idn  Imc1  Imc2  InverseVariance  JointAverage  JointEnergy  JointEntropy  MCC  MaximumProbability  SumAverage  SumEntropy  SumSquares |

| Gray Level Dependence  Matrix(GLDM) Features | 57  58  59  60  61  62  63  64  65  66  67  68  69  70 | DependenceEntropy  DependenceNonUniformity  DependenceNonUniformityNormalized  DependenceVariance  GrayLevelNonUniformity GrayLevelVariance  HighGrayLevelEmphasis  LargeDependenceEmphasis  LargeDependenceHighGrayLevelEmphasis LargeDependenceLowGrayLevelEmphasis  LowGrayLevelEmphasis  SmallDependenceEmphasis  SmallDependenceHighGrayLevelEmphasis SmallDependenceLowGrayLevelEmphasis |
| --- | --- | --- |
| Gray Level Run Length  Matrix(GLRLM) Features | 71  72  73  74  75  76  77  78  79  80  81  82  83  84  85  86 | GrayLevelNonUniformity  GrayLevelNonUniformityNormalized GrayLevelVariance  HighGrayLevelRunEmphasis LongRunEmphasis  LongRunHighGrayLevelEmphasis LongRunLowGrayLevelEmphasis LowGrayLevelRunEmphasis  RunEntropy  RunLengthNonUniformity  RunLengthNonUniformityNormalized RunPercentage  RunVariance  ShortRunEmphasis  ShortRunHighGrayLevelEmphasis ShortRunLowGrayLevelEmphasis |
| Gray Level Size Zone Matrix (GLSZM) Features | 87  88  89  90  91  92  93  94  95  96  97  98  99  100  101  102 | GrayLevelNonUniformity  GrayLevelNonUniformityNormalized GrayLevelVariance  HighGrayLevelZoneEmphasis LargeAreaEmphasis  LargeAreaHighGrayLevelEmphasis LargeAreaLowGrayLevelEmphasis LowGrayLevelZoneEmphasis  SizeZoneNonUniformity  SizeZoneNonUniformityNormalized  SmallAreaEmphasis  SmallAreaHighGrayLevelEmphasis SmallAreaLowGrayLevelEmphasis  ZoneEntropy  ZonePercentae ZoneVariance |
| Neighbouring Gray Tone Difference Matrix (NGTDM) Features | 103  104  105  106  107 | Busyness  Coarsenes Complexity Contrast  Strength |

**Supplementary Materials 4: Stable Feature Selection Methodology for Radiomics Analysis**

The stability assessment protocol integrates a multi-stage analytical pipeline designed to ensure reproducible identification of robust radiomic features across heterogeneous datasets. At the data preparation stage, input features undergo systematic quality control beginning with automated type normalization. All non-numeric entries are first converted through strict type casting, with conversion failures mapped to standardized missing value flags, ensuring mathematical operability while preserving data structure. This is followed by a two-phase missing value resolution protocol wherein features exceeding 20% missing instances are eliminated to prevent bias amplification, while remaining gaps in retained features are addressed through iterative forward-backward filling sequences that account for cohort-level information continuity. Subsequent outlier mitigation employs quantile-based value capping, constraining extreme observations to the 1st and 99th percentiles of their empirical distributions, thus preserving ordinal relationships while reducing sensitivity to acquisition anomalies.

Feature stability quantification employs a composite evaluation framework addressing dispersion, distributional, and contextual consistency. Initial variance screening retains only those features demonstrating biologically plausible variation through sequential thresholding—first requiring adequate dispersion in pooled data aggregates, then validating minimum variation thresholds within individual cohorts. Distributional concordance is assessed through paired comparisons of interquartile concentration patterns quantified via IQR ratio exponential transforms and nonparametric Kolmogorov-Smirnov divergence measures. Contextual consistency monitoring tracks standardized mean differences across datasets through Cohen's effect size normalization, establishing safeguards against systematic cohort shifts.

Implementation rigor is maintained through software design principles ensuring full computational replicability. All analytical thresholds and control parameters—including variance boundaries (10^-6 to 10^-8), outlier capping ranges (1%-99%), and stability scoring coefficients (variance 40%, IQR 30%, K-S 20%, effect size 10%)—are encapsulated in version-controlled configuration files separate from core logic. Execution environments are precisely constrained through frozen dependency trees and containerized runtime stacks cross-validated for Windows/Linux parity. Comprehensive provenance tracking captures dataset checksums, parameter snapshots, and intermediate state dumps at each analytical checkpoint. Standardized output packages include comparative distribution trajectory plots highlighting salient feature stabilities (Figure S2).


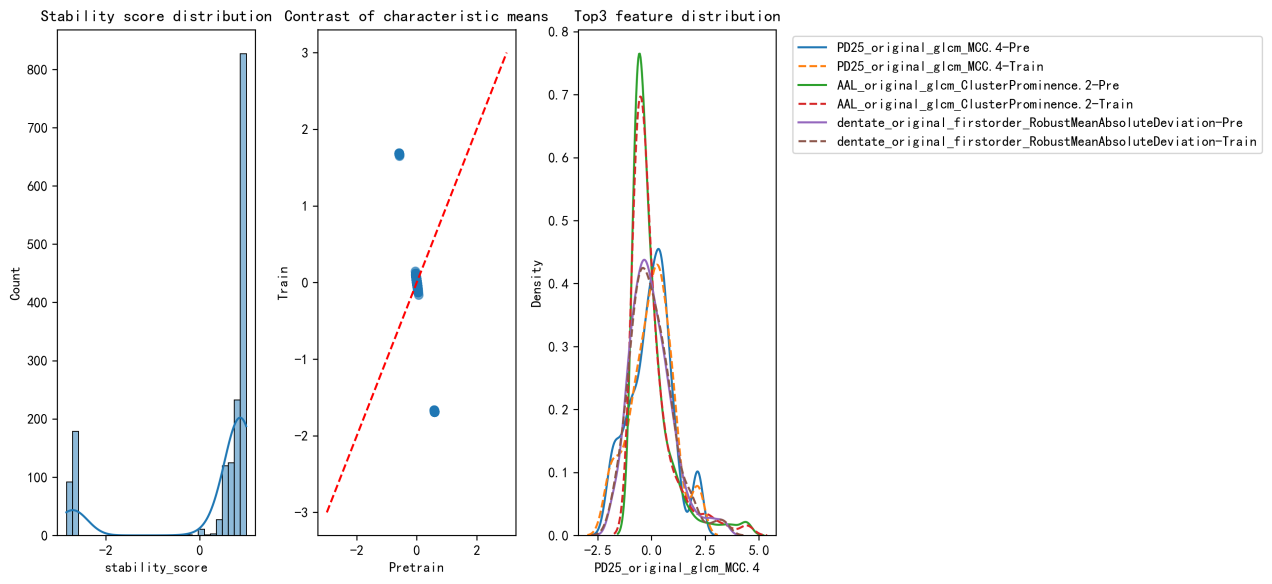


**Figure S2**: plot of highlighting salient feature stabilities

Eight ROIs corresponding to LASSO-selected radiomic features were visualized using BrainNet Viewer software on the MNI brain template. Anatomical regions were labeled based on AAL3 atlas and include the right putamen, caudate nucleus, bilateral superior frontal gyri, supplementary motor areas, and occipital gyri, indicating strong neuroanatomical relevance of the selected features.


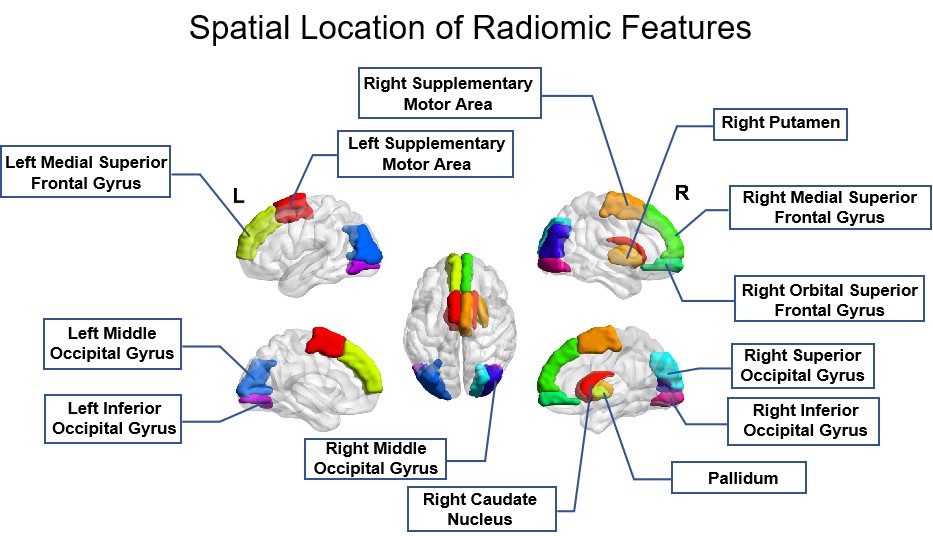


**Figure S3**: Spatial localization of selected radiomic features

**Supplementary Materials 5: Performance of model selection**

| **Cohort** | **Method** | **Accuracy (%)** | **AUC score (%)** | **F1 score (%)** | **Recall (%)** | **Precision (%)** |
| --- | --- | --- | --- | --- | --- | --- |
| Pre training Cohort | DenseNet | 0.919 ± 0.021 | 0.941 ± 0.002 | 0.895 ± 0.052 | 0.910 ± 0.020 | 0.900 ± 0.038 |
|  | **DNN** | **0.929 ± 0.023** | **0.963 ± 0.009** | **0.905 ± 0.054** | **0.923 ± 0.017** | **0.912 ± 0.037** |
|  | ResNet 50 | 0.894 ± 0.015 | 0.939 ± 0.004 | 0.847 ± 0.109 | 0.886 ± 0.035 | 0.863 ± 0.073 |
|  | GoogleNet | 0.894 ± 0.017 | 0.941 ± 0.005 | 0.841 ± 0.118 | 0.900 ± 0.040 | 0.864 ± 0.074 |

The results from the improved DNN extractor selection are summarized in Table S4, while the experimental outcomes from the traditional machine learning approaches are detailed in Table S5. The diagnostic capability of the RDDNN model was evaluated on both the blind-test set from the blind-test cohort and an additional test set from Germany. Notably, the RDDNN model outperformed radiomics alone, demonstrating enhanced efficacy in diagnostic performance.

**Table S4**: Pretraining performance of each traditional DNN model

*DNN:* deep neural network (based on ResNet34)

**Table S5**: Classification performance of each machine learning method

| **Cohort** | **Method** | **Accuracy (%)** | **AUC score (%)** | **F1 score (%)** | **Recall (%)** | **Precision (%)** |
| --- | --- | --- | --- | --- | --- | --- |
| Pre  training Cohort | **XGBoost** | **0.891 ± 0.028** | **0.960 ± 0.015** | **0.865 ± 0.053** | **0.855 ± 0.071** | **0.882 ± 0.046** |
|  | Adaboost | 0.887 ± 0.022 | 0.953 ± 0.016 | 0.859 ± 0.055 | 0.842 ± 0.080 | 0.875 ± 0.027 |
|  | RF | 0.889 ± 0.027 | 0.960 ± 0.016 | 0.861 ± 0.060 | 0.849 ± 0.081 | 0.874 ± 0.043 |
|  | SVM | 0.877 ± 0.019 | 0.948 ± 0.014 | 0.850 ± 0.055 | 0.845 ± 0.068 | 0.861 ± 0.039 |
|  | LR | 0.859 ± 0.036 | 0.873 ± 0.034 | 0.830 ± 0.060 | 0.823 ± 0.070 | 0.842 ± 0.057 |
| Training Cohort | **XGBoost** | **0.937 ± 0.023** | **0.986 ± 0.008** | **0.919 ± 0.039** | **0.916 ± 0.049** | **0.925 ± 0.026** |
|  | Adaboost | 0.928 ± 0.020 | 0.987 ± 0.005 | 0.912 ± 0.032 | 0.908 ± 0.047 | 0.920 ± 0.015 |
|  | RF | 0.936 ± 0.032 | 0.987 ± 0.008 | 0.918 ± 0.043 | 0.917 ± 0.043 | 0.920 ± 0.041 |
|  | SVM | 0.934 ± 0.028 | 0.987 ± 0.007 | 0.917 ± 0.042 | 0.914 ± 0.055 | 0.923 ± 0.028 |
|  | LR | 0.921 ± 0.028 | 0.967 ± 0.011 | 0.906 ± 0.031 | 0.901 ± 0.039 | 0.914 ± 0.030 |

*RF,* RandomForest*;*

*SVM,* Support Vector Machine*;*

*LR,* LogisticRegression;

**Supplementary Materials 6: Layer-CAM and DeepLIFT**

To enhance interpretability, we incorporated additional voxel-level visualizations using Layer-CAM and DeepLIFT methods for both the CNN-based local channel and Transformer-based global channel, as illustrated in Supplementary Figures S4 and S5.

DeepLIFT is a gradient-free interpretability technique that quantifies the contribution of each input voxel to the model output by comparing the activation difference from a reference input. Unlike gradient-based methods, DeepLIFT avoids saturation issues and provides more stable and interpretable relevance maps.

In this study, DeepLIFT was applied to both the local (CNN-based) and global (Transformer-based) channels. All computations were implemented using the Captum library under the PyTorch framework. For consistency with Layer-CAM analysis, we selected the 27th sagittal slice of each subject as the representative plane for 2D visualization. This slice was chosen to optimize the visibility of core brain structures, including the basal ganglia and midbrain.

Figure S4 demonstrates Layer-CAM activations across multiple layers. In the CNN architecture, stronger responses emerged in deeper layers (Layer_3 and Layer_4), with MSA and PSP groups exhibiting pronounced heatmap intensity in the midbrain and striatal regions, consistent with known pathological patterns. In the Transformer model, although activations were more diffuse, consistent focus appeared in PSP and IPD cases over the thalamus and midbrain—reinforcing the channel's disease relevance.

Figure S5 shows DeepLIFT-based saliency maps generated via the Captum library. In CNNs, local channel saliency was spatially broad yet layer-consistent, while in Transformers, the global channel highlighted key regions such as the frontal cortex and basal ganglia. These results further support the structural convergence of both models in identifying disease-relevant features through complementary mechanisms.


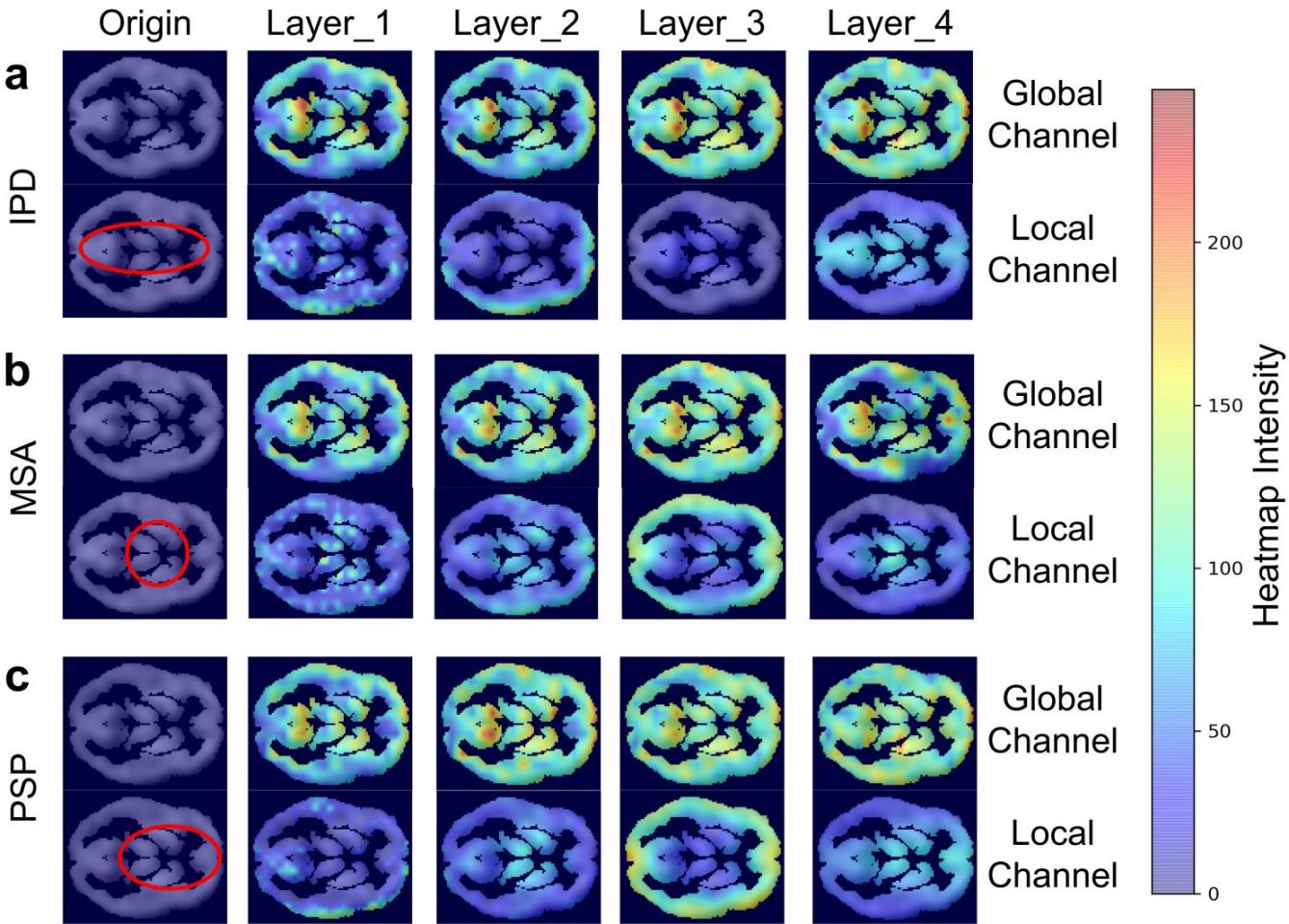


**Figure S4:** Layer-wise voxel-level attention maps using Layer-CAM for DNN and Transformer channels.(a-c) show the global (Transformer-based) and local (DNN-based) attention maps across four network layers for representative IPD, MSA, and PSP cases, respectively.


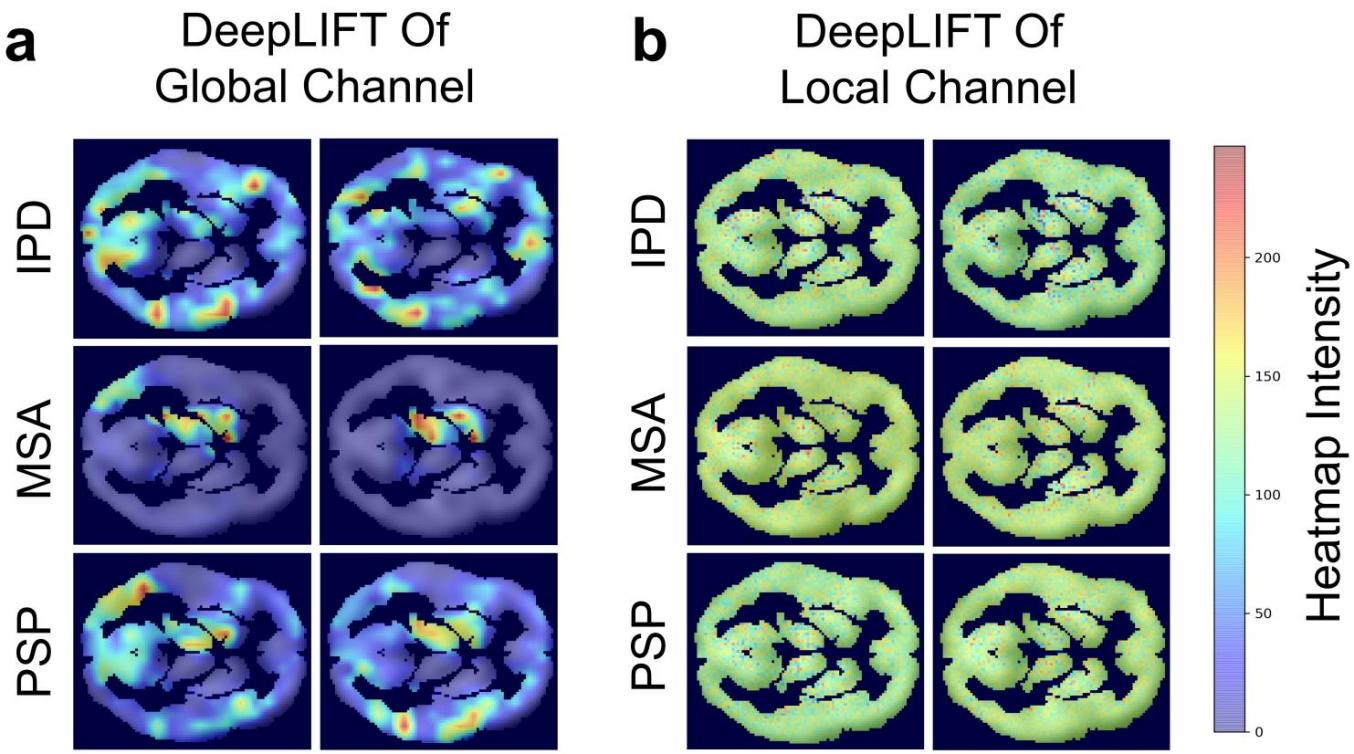


**Figure S5:** DeepLIFT-based attribution maps for global and local channels in CNN and ViT architectures. Voxel-level saliency maps were computed using the DeepLIFT algorithm implemented via Captum. (a) global-channel attribution patterns across three representative disease groups (IPD, MSA, PSP) (b) local-channel attribution patterns across three representative disease groups (IPD, MSA, PSP).

**Supplementary Materials 7: SHAP Value consistency across both cohorts**

Hive plots depict the distribution of SHAP values for top contributing biomarkers in the differentiation of MSA, PSP, and IPD subtypes across two test cohorts (Cohort A: blind-test cohort, B: additional test cohort). Each hexagonal cell represents a feature, with horizontal spread indicating SHAP value magnitude and vertical jitter reducing overplotting. The near-identical dispersion patterns between cohorts for all disease classes (e.g.[Global_latent_43] in MSA) demonstrate high cross-cohort consistency in model interpretation.


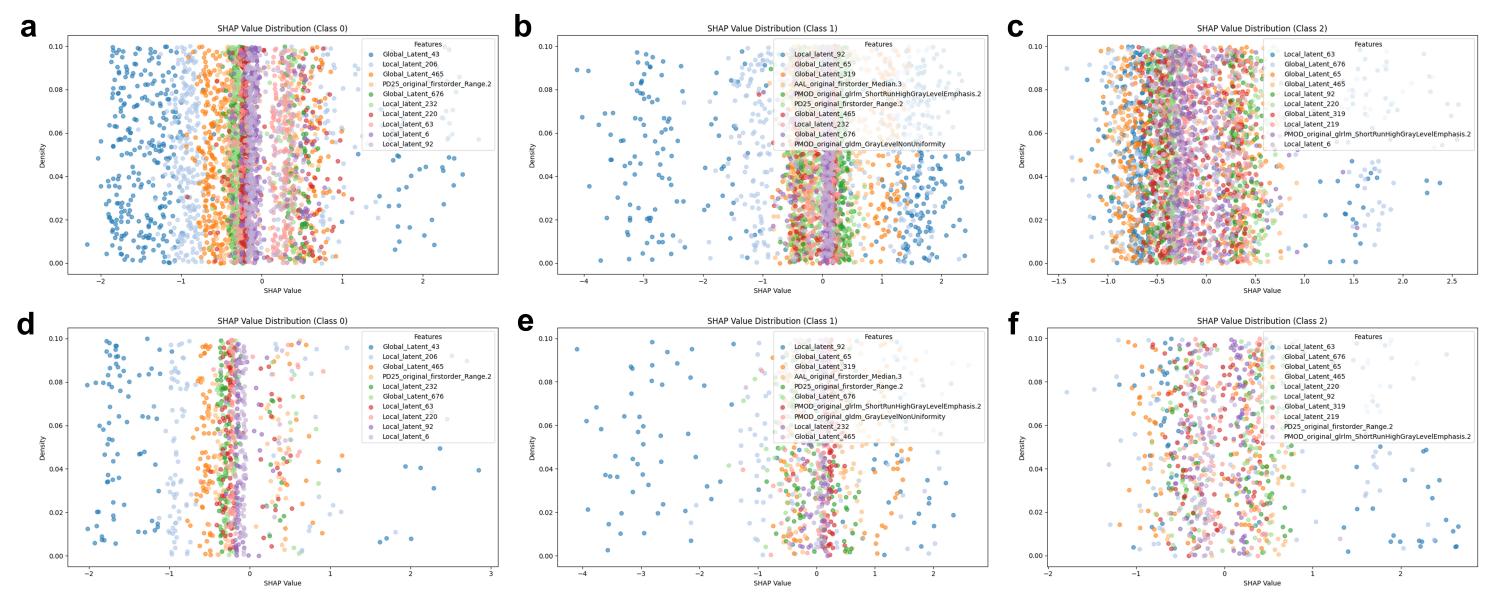


**Figure S6**: Hive Plot visualization of SHAP Value consistency across two test cohorts

**Supplementary Materials 8: t-SNE Visualization Methodology**

For systematic evaluation of feature stability across dual test cohorts, we implemented t-distributed stochastic neighbor embedding (t-SNE) visualization through the following protocol:

1. Feature Extraction Layer Selection:

Local Channel: Activated features from the final dilated convolutional layer (128 filters, dilation rate=3) were sampled at 79×95×69 resolution, capturing hierarchical receptive field patterns.

Global Channel: Transformer-derived patch embeddings (dimension 768) from the encoder's final self-attention layer were concatenated with global average pooling outputs.

1. Feature Fusion & Normalization:

Multi-scale features were projected into shared latent space using channel-wise L2 normalization.

Dimensionality reduction via PCA retained 95% variance before t-SNE implementation.

1. Parameter Configuration:

Performed Barnes-Hut approximation with perplexity=30, learning rate=200, and 1000 iterations and dual test set visualizations used identical random seeds (seed=42) for reproducibility.


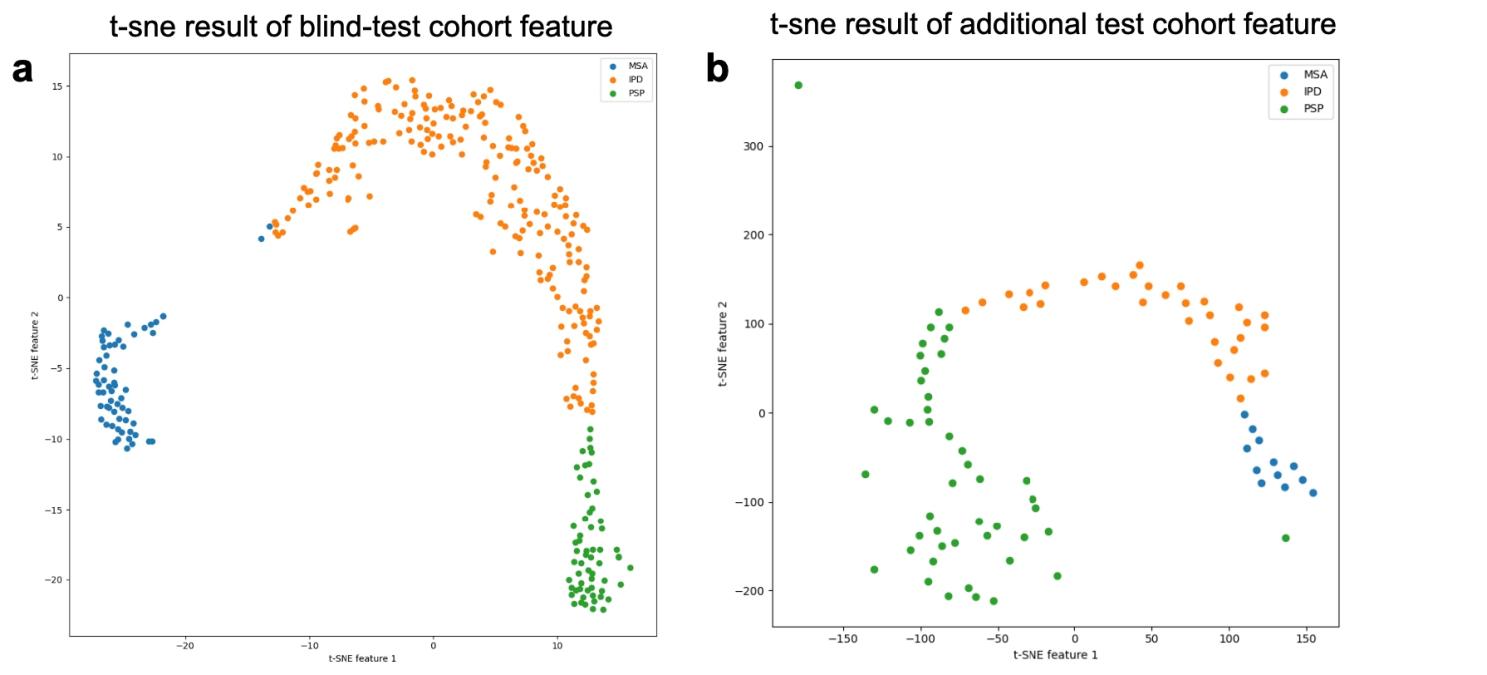


**Figure S7**: t-SNE Visualization of blind-test cohort and additional test cohort

Supplementary Figure S7 presents t-SNE plots of the learned DNN features for the blind-test cohort (a) and the additional test cohort (b). These visualizations offer insights into the feature separability and clustering structure learned by the model across cohorts.

In Figure S7a, a clear separation can be observed among the three diagnostic groups (MSA, IPD, PSP), with each forming compact and distinct clusters. This indicates that the model successfully learned discriminative representations capable of distinguishing disease subtypes in the blind-test data.

In Figure S7b, although there is slightly more overlap between clusters—particularly between IPD and PSP—most samples still follow a similar arc-like distribution, preserving relative inter-class relationships. This suggests that the learned features are stable and transferable, demonstrating generalizability across different populations and imaging platforms.

Overall, these t-SNE plots visually support the model's capacity to extract meaningful latent features with strong discriminative power and cohort-level consistency.

**Supplementary Materials 9: Cross-Cohort Pearson Correlation of Key Latent Features With UPDRS-III**

To further validate the clinical relevance of RDDNN-derived features, we performed Pearson correlation analyses between top-ranking fusion features and UPDRS-III scores within each cohort. Supplementary Figure S4 presents the correlation results for three key latent features—Global_latent_361, Local_latent_249, and Global_latent_365—in both the Chinese and additional test cohorts.

Global_latent_361 exhibited a consistent positive correlation with UPDRS scores (r = 0.17, p < 0.005 in the blind-test cohort; r = 0.35, p < 0.005 in the additional test cohort). Similarly, Local_latent_249 also showed stable positive associations (r = 0.16 and r = 0.33, respectively; both p < 0.005). In contrast, Global_latent_365 demonstrated a significant negative correlation with motor symptom severity, with coefficients of r = –0.17 (p < 0.005) in the blind-test cohort and r = –0.29 (p < 0.005) in the additional test cohort. These findings support the interpretability of the selected latent features in reflecting clinically meaningful motor dysfunction patterns across populations.


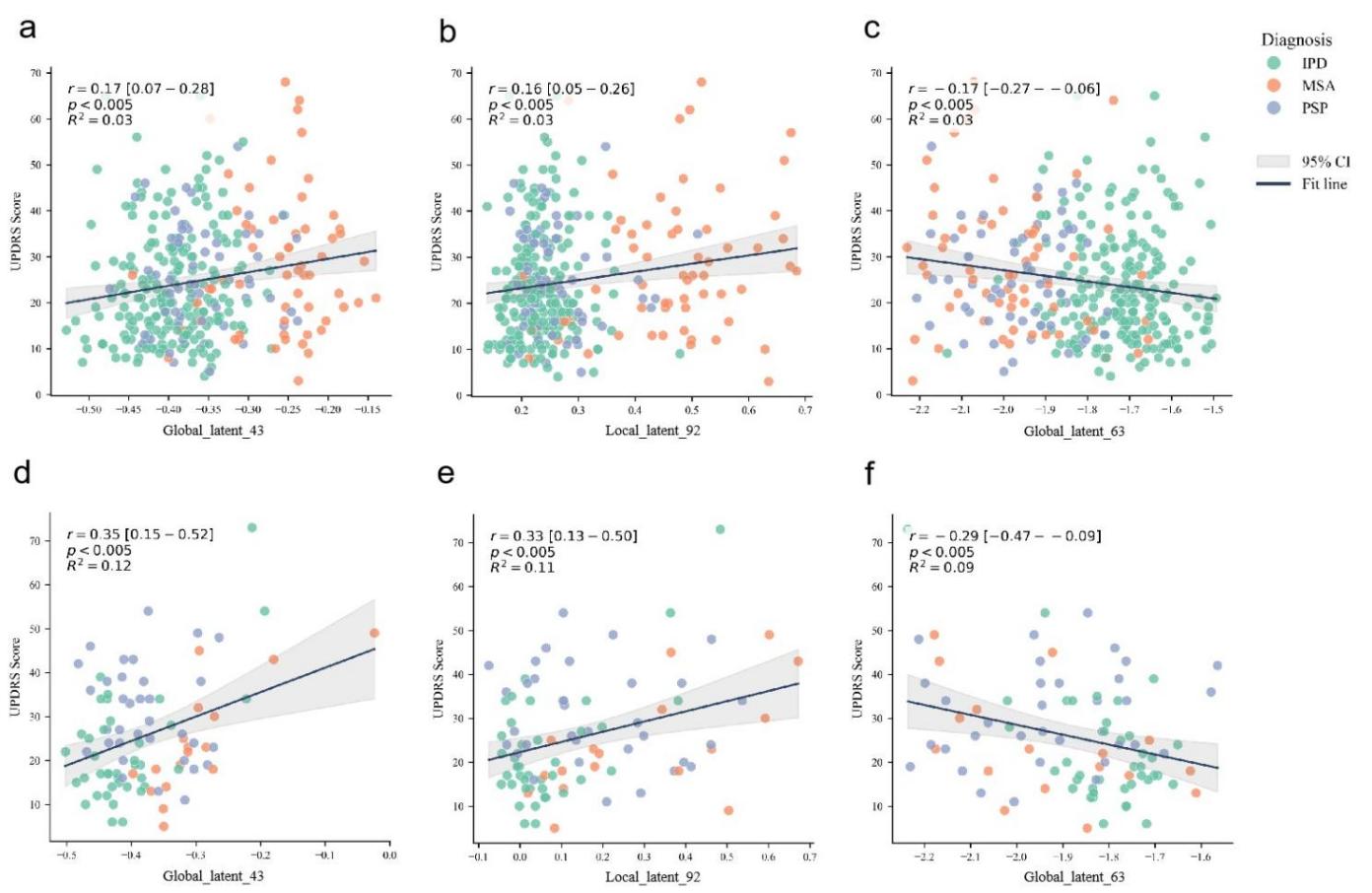


**Figure S8：**Pearson correlation analysis between representative latent features and UPDRS III scores in both cohorts. (a–c) Correlation between Global_latent_43,Local_latent_92, and Global_latent_63 with UPDRSIII scores in the blind-test cohort.(d–f) Correlation between the same features and UPDRSIII scores in the additional test cohort.

Each dot represents an individual subject, color-coded by diagnosis (IPD, MSA, PSP). Solid lines denote linear regression fit with 95% confidence intervals. All correlations are statistically significant (p < 0.005), with Global_latent_43 and Local_latent_92 showing positive associations and Global_latent_63 showing a negative association with motor symptom severity.

**REFERENCES**

**1.** Höglinger GU, Respondek G, Stamelou M, et al. Clinical diagnosis of progressive supranuclear palsy: the movement disorder society criteria. *Mov Disord.* 2017;32:853-864.

**2.** Postuma RB, Berg D, Stern M, et al. MDS clinical diagnostic criteria for Parkinson's disease. *Mov Disord.* 2015;30:1591-1601.

**3.** Gilman S, others. Second consensus statement on the diagnosis of multiple system atrophy. *Neurology.* 2008;71:670-676.

**4.** Litvan I, Agid Y, Calne D, et al. Clinical research criteria for the diagnosis of progressive supranuclear palsy (Steele-Richardson-Olszewski syndrome) report ofthe NINDS-SPSP international workshop. *Neurology.* 1996;47:1-9.

**5.** Hughes AJ, Daniel SE, Kilford L, Lees AJ. Accuracy of clinical diagnosis ofidiopathic Parkinson's disease: a clinico-pathological study of 100 cases. *JNeurol Neurosurg Psychiatry.* 1992;55:181-184.
